# The *Pseudomonas aeruginosa* homeostasis enzyme AlgL clears the periplasmic space of accumulated alginate during polymer biosynthesis

**DOI:** 10.1016/j.jbc.2021.101560

**Published:** 2022-01-03

**Authors:** Andreea A. Gheorghita, Francis Wolfram, Gregory B. Whitfield, Holly M. Jacobs, Roland Pfoh, Steven S.Y. Wong, Allison K. Guitor, Mara C. Goodyear, Alison M. Berezuk, Cezar M. Khursigara, Matthew R. Parsek, P. Lynne Howell

**Affiliations:** 1 Program in Molecular Medicine, The Hospital for Sick Children, Toronto, Ontario, Canada; 2 Department of Biochemistry, University of Toronto, Toronto, Ontario, Canada; 3 Molecular and Cellular Biology Graduate Program, University of Washington, Seattle, Washington, USA; 4 Department of Molecular and Cellular Biology, University of Guelph, Guelph, Ontario, Canada; 5 Department of Microbiology, University of Washington, Seattle, Washington, USA

**Keywords:** *Pseudomonas aeruginosa*, alginate lyase, biofilm, polysaccharide, bacterial genetics, crystallography, enzyme structure, structure–function, Carb, carbenicillin, CAZy, Carbohydrate-Active enZYmes, CF, cystic fibrosis, E.C., enzyme commission, eDNA, extracellular DNA, Gen, gentamicin, GulA, L-guluronic acid/guluronate, HRP, horseradish peroxidase, IPTG, isopropyl β-D-1-thiogalactopyranoside, Kan, kanamycin, *kcat*, turnover number, LB, lysogeny broth, MAP, modified alginate producing, ManA_3_, mannuronate trisaccharide, ManA, D-mannuronic acid/mannuronate, NSLB, no-salt lysogeny broth, OD, optical density, PL, polysaccharide lyase, polyM, polymannuronate, polyMG, polymannuronate-guluronate, PNAG, poly-*N*-acetylglucosamine, SAD, single-wavelength anomalous diffraction, SeMet, selenomethionine, TBS, tris-buffered saline, TBST, tris-buffered saline with Tween-20, TEM, transmission electron microscopy, VBMM, Vogel–Bonner minimal medium, VSV-G, vesicular stomatitis virus glycoprotein

## Abstract

*Pseudomonas aeruginosa* is an opportunistic human pathogen and a leading cause of chronic infection in the lungs of individuals with cystic fibrosis. After colonization, *P. aeruginosa* often undergoes a phenotypic conversion to mucoidy, characterized by overproduction of the alginate exopolysaccharide. This conversion is correlated with poorer patient prognoses. The majority of genes required for alginate synthesis, including the alginate lyase, *algL*, are located in a single operon. Previous investigations of AlgL have resulted in several divergent hypotheses regarding the protein’s role in alginate production. To address these discrepancies, we determined the structure of AlgL and, using multiple sequence alignments, identified key active site residues involved in alginate binding and catalysis. *In vitro* enzymatic analysis of active site mutants highlights R249 and Y256 as key residues required for alginate lyase activity. In a genetically engineered *P. aeruginosa* strain where alginate biosynthesis is under arabinose control, we found that AlgL is required for cell viability and maintaining membrane integrity during alginate production. We demonstrate that AlgL functions as a homeostasis enzyme to clear the periplasmic space of accumulated polymer. Constitutive expression of the AlgU/T sigma factor mitigates the effects of an *algL* deletion during alginate production, suggesting that an AlgU/T-regulated protein or proteins can compensate for an *algL* deletion. Together, our study demonstrates the role of AlgL in alginate biosynthesis, explains the discrepancies observed previously across other *P. aeruginosa* Δ*algL* genetic backgrounds, and clarifies the existing divergent data regarding the function of AlgL as an alginate degrading enzyme.

Biofilms are highly structured communities of bacterial cells embedded in a self-produced matrix (1, 2). Their ability to adhere to a variety of biotic and abiotic surfaces makes biofilms ubiquitous in natural, industrial, and clinical settings. Biofilms are found in deep-sea vents, freshwater rivers, drinking water distribution systems, food processing equipment, and even the International Space Station and are responsible for the contamination of medical devices such as catheters, prosthetic heart valves, and cardiac pacemakers (3, 4, 5, 6, 7, 8). Biofilms are also responsible for tissue-related infections including diffuse panbronchiolitis, lung infections in individuals with cystic fibrosis (CF), and chronic wound infections (9, 10, 11, 12, 13, 14). Composed of proteins, extracellular DNA (eDNA), and exopolysaccharides, the biofilm matrix confers an advantage to the bacteria by providing protection from antibiotic treatments and the host’s immune response (3, 15). The opportunistic human pathogen *Pseudomonas aeruginosa* is notorious for establishing chronic infections in the lungs of individuals with CF and tolerating antibiotic treatment through formation of a biofilm.

*P. aeruginosa* is genetically capable of producing three distinct exopolysaccharides as part of its biofilm matrix: Pel, Psl, and alginate. Each polymer plays an important role in chronic infections (16). For example, Pel binds to eDNA in the stalk of the biofilm near the point of attachment, is associated with *P. aeruginosa* aggregates in CF sputum, and provides protection from aminoglycoside antibiotics (17, 18, 19). Psl has also been associated with *P. aeruginosa* aggregates in CF sputum and is important for surface adhesion and biofilm structure (19, 20, 21), while alginate is typically associated with the chronic lung infections suffered by individuals with CF where it blocks cell-mediated phagocytosis and hence aids in evasion of the host immune response (22, 23). Alginate is produced when *P. aeruginosa* converts to a mucoid state (24). This conversion is typically induced by a mutation in the antisigma factor MucA that is responsible for regulating the sigma factor AlgU/T (25, 26). Alginate overproduction also promotes *P. aeruginosa* coinfection with *Staphylococcus aureus*, thus influencing CF patient outcomes as coinfection is associated with decreased lung function (27, 28, 29). Upregulation of genes involved in alginate biosynthesis has also been observed in a *P. aeruginosa* murine burn wound model, demonstrating the polymer’s importance in biofilm formation outside of the CF lung environment (30).

Except for *algC*, the genes required for alginate biosynthesis are clustered in a single operon (31) (Fig. S1). Alginate is initially synthesized as an anionic homopolymer that is chemically modified by acetylation and epimerization in the periplasm prior to the export of the polymer (Fig. S1). Proteins involved in this process are hypothesized to form a multiprotein complex that spans the inner and outer membranes. AlgA, AlgC, and AlgD are involved in the production of the alginate precursor molecule GDP-mannuronic acid, which, in response to cyclic di-GMP binding to the PilZ domain of Alg44, is polymerized by Alg8 and Alg44 to form β-1,4-linked D-mannuronic acid (ManA) (31, 32, 33, 34, 35). Once in the periplasm, AlgF, AlgI, AlgJ, and AlgX acetylate the ManA homopolymer at the O2 and/or O3 hydroxyls (31, 36, 37, 38), while AlgG selectively epimerizes nonacetylated ManA residues to L-guluronate (GulA) (31). The polymer is exported from the cell *via* AlgK and the outer membrane ß-barrel porin AlgE (39, 40).

The *alg* operon also encodes a periplasmic lyase, AlgL. Characterization of this enzyme *in vitro* demonstrated that it preferentially degrades nonacetylated polymannuronate (polyM) *via* a ß-elimination mechanism (41, 42). Despite our detailed understanding of its reaction mechanism, the function of AlgL in alginate biosynthesis remains poorly understood. Several different roles for the enzyme have been proposed. These include the regulation of the length of secreted alginate polymer by cleaving β-1,4-linkages between ManA residues prior to export (43, 44); aiding in biofilm detachment (45); and degradation of alginate that is not exported from the cell to prevent its accumulation within the periplasmic space (46, 47). Studies have also suggested that the enzyme is part of a multiprotein complex with AlgG, AlgX, and AlgK and that it assists in transporting the polymer across the outer membrane (47). This contrasts with more recent studies that suggest AlgL does not associate with other alginate biosynthesis proteins (48). Most notably, AlgL has variably been suggested to be required for alginate production (49), required for *P. aeruginosa* viability during alginate production (47), and completely dispensable for alginate production and biofilm biomass (48).

In the present study, we employed a multidisciplinary approach to address these discrepancies and determine the role of AlgL. Our structure of wild-type (WT) *P. aeruginosa* AlgL in complex with ManA, its comparison with other bacterial alginate lyases, and *in vitro* enzyme kinetic analyses have enabled the identification of active site residues important for alginate binding and catalysis. In a genetically engineered strain where alginate biosynthesis can be controlled using arabinose, we demonstrate that absence of *algL* or mutation of key catalytic residues is detrimental for *P. aeruginosa* growth during alginate biosynthesis and results in abnormal cellular morphology. Furthermore, we show that AlgL prevents lethal accumulation of alginate during polymer production suggesting that the enzyme is important for homeostasis of the periplasm. Finally, using a *mucA22 P. aeruginosa* background, we found that absence of *algL* is tolerated. Taken together, our data suggest that, when necessary, AlgL functions as a periplasmic homeostasis enzyme during alginate production and that a protein or proteins can compensate for its loss when the entire AlgU/T regulon is upregulated.

## Results

### AlgL has an (α/α)_5_ toroid fold

**Figure 1:**
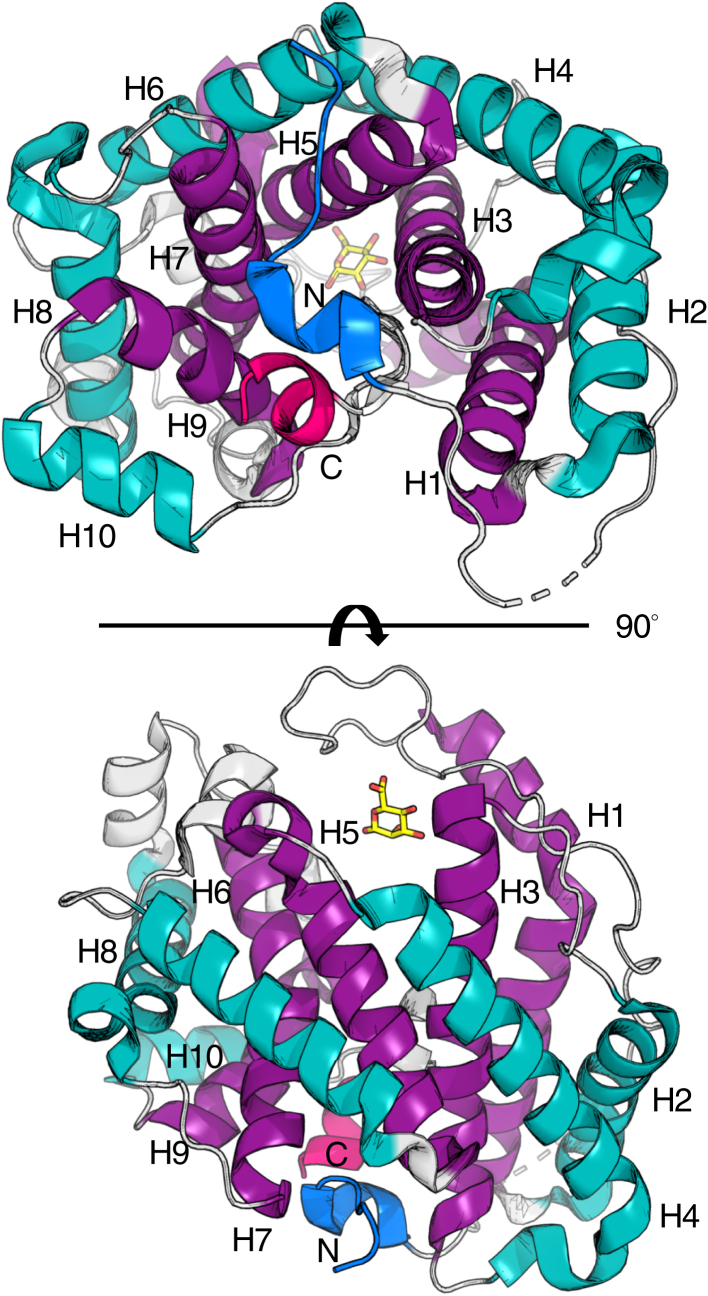
**Structure of AlgL from *Pseudomonas aeruginosa* reveals an (α/α)**_**5**_**toroid fold with five pairs of antiparallel α-helices.** Helices are numbered from H1 to H10. Outer helices are colored in *teal*, inner helices are colored in *purple*, the N terminus is colored in *blue*, and the C terminus is colored in *magenta*. A single monomer of mannuronate (ManA) (*yellow*) is observed.

To enable our functional *in vivo* studies of AlgL and delineate the role of this enzyme in alginate biosynthesis, we first determined its structure, minus its signal sequence (NHis_6_-AlgL^28–362^), to 1.65 Å resolution using selenomethionine (SeMet) incorporation and the single-wavelength anomalous dispersion (SAD) technique. AlgL crystallized in space group *P*2_1_2_1_2_1_ with one molecule in the asymmetric unit (Table 1). The final model of AlgL was refined to a final R_work_ and R_free_ of 17.7% and 19.4%, respectively. The structure reveals that AlgL adopts an (α/α)_5_ toroid fold with five pairs of antiparallel α-helices (Fig. 1). Examination of the carbohydrate-active enzymes (CAZy; http://www.cazy.org) database reveals that this (α/α)_n_ spatial arrangement has been reported previously in alginate lyases from the polysaccharide lyase (PL) family PL-5 to which AlgL belongs, as well as 12 other PL families (50, 51).

**Table 1:** Data collection and refinement statistics

	AlgL SeMet	AlgL	AlgL H202A	AlgL K66A
Data collection				
Wavelength (Å)	0.97920	1.075	1.075	1.5406
Temperature (K)	100	100	100	100
Space Group	*P*2_1_2_1_2_1_	*P*2_1_2_1_2_1_	*P*2_1_2_1_2_1_	*P*2_1_2_1_2
Cell Dimensions				
*a*, *b*, *c* (Å)	56.0, 59.8, 94.6	56.4, 59.6, 102.1	56.4, 59.4, 102.1	67.5, 58.7, 76.2
*α*, *β*, *γ* (°)	90, 90, 90	90, 90, 90	90, 90, 90	90, 90, 90
Resolution (Å)	50.0–2.1 (2.18–2.10)	50.0–1.64 (1.70–1.64)	50.0–1.64 (1.70–1.64)	20.3–2.50 (2.60–2.50)
Total No. of Reflections	253,565	542,308	594,045	127,670
No. of Unique Reflections	19,041	43,027	43,079	10,903
Redundancy	13.4 (10.5)	13.2 (10.6)	15.0 (12.8)	10.3 (7.75)
Completeness (%)		95.2 (71.2)	99.0 (94.0)	99.3 (100.0)
Average *I*/σ (*I*)	14.2 (2.5)	25.8 (3.2)	22.0 (11.6)	16.0 (3.3)
R_merge_^a^ (%)	16.5 (65.1)	8.9 (55.6)	9.4 (26.2)	9.8 (43.5)
Refinement^b^				
R_work_/R_free_ (%)^c^		17.7/19.4	16.8/19.3	24.6/26.9
No. atoms				
Protein		2601	2606	2458
Ligand		13		
Solvent		107	161	38
Average B-factors (Å^2^)				
Protein		25.0	22.1	32.8
Ligand		48.2		
Water		25.0	25.9	35.0
Root mean square deviations				
Bond lengths (Å)		0.007	0.006	0.007
Bond angles (°)		0.97	0.98	1.5
Ramachandran plot^d^				
Total favored (%)		99.0	99.0	97.0
Total allowed (%)		100	100	100
Est. coordinate error (Å)^e^		0.16	0.12	0.30
PDB code		4OZV	4OZW	7SA8

Values in parentheses are for the highest-resolution shell.

a *R*_merge_ = Σ_hkl_ Σ_i_ |*I*_*i*_*(hkl)* – <*I(hkl)*>|/Σ_hkl_ Σ_i_ I_i_(hkl), where *I*_*i*_(*hkl*) and <*I*(*hkl*)> represent the diffraction-intensity values of the individual measurements and the corresponding mean values, respectively.

b AlgL and AlgL H202A were refined using PHENIX.REFINE (95); AlgL K66A was refined using REFMAC5 (99).

c *R*_work_ = Σ||F_obs_| − k|F_calc_||/|F_obs_|, where F_obs_ and F_calc_ are the observed and calculated structure factors, respectively. R_free_ is the sum extended over a subset of reflections (5%) excluded from all stages of the refinement.

d As calculated using MolProbity (114).

e Maximum-Likelihood-Based Coordinate Error as determined by PHENIX (95) for AlgL and AlgL H202A. Estimated Overall Coordinate Error Based on Maximum Likelihood as determined by REFMAC5 (99) for AlgL K66A.

**Figure 2:**
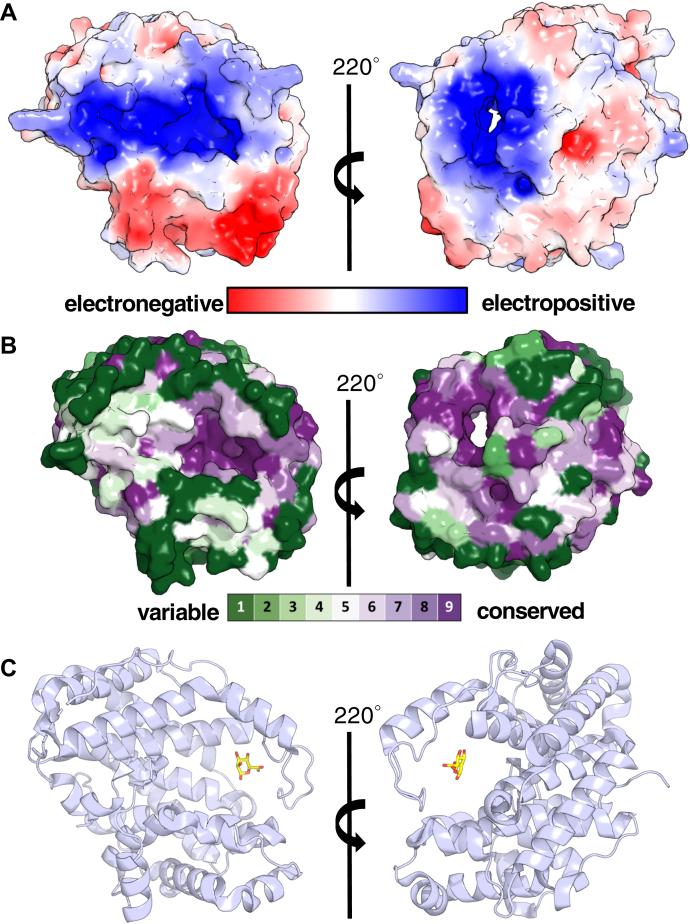
**Structure of *Pseudomonas aeruginosa* AlgL reveals an elongated, highly conserved, and strongly electropositive groove.***A*, electrostatic surface representation of AlgL calculated by APBS Tools; contoured from +5 (*blue*) to −5 (*red*) kT/e (102). *B*, conservation surface representation of AlgL calculated by the ConSurf server; *green* indicates residues that are less conserved, and *purple* indicates residues that are highly conserved (103). *C*, AlgL in complex with a mannuronate residue (*yellow*) reveals a partially enclosed structure.

Although we cocrystallized the protein with a mannuronate trisaccharide (ManA_3_), we were only able to detect electron density for a sugar monomer suggesting perhaps that cleavage of the ManA_3_ substrate may have occurred *in crystallo* or that the remaining sugar units/moieties were disordered in the structure (Fig. 1). While this ManA identifies the location of the active site, we anticipate that it will contain multiple sugar-binding sites given that AlgL’s activity increases linearly with the number of residues in the substrate (41). Indeed, examination of the enzyme’s surface electrostatic properties and residue conservation revealed an elongated, highly conserved, and strongly electropositive groove (Fig. 2, *A* and *B*). In addition to the pronounced, long substrate-binding groove, we also identified an extended loop that partially closes over the ManA-binding site, known as the lid-loop (Fig. 2*C*).

### Structural comparisons identify AlgL residues required for alginate binding and catalysis

**Figure 3:**
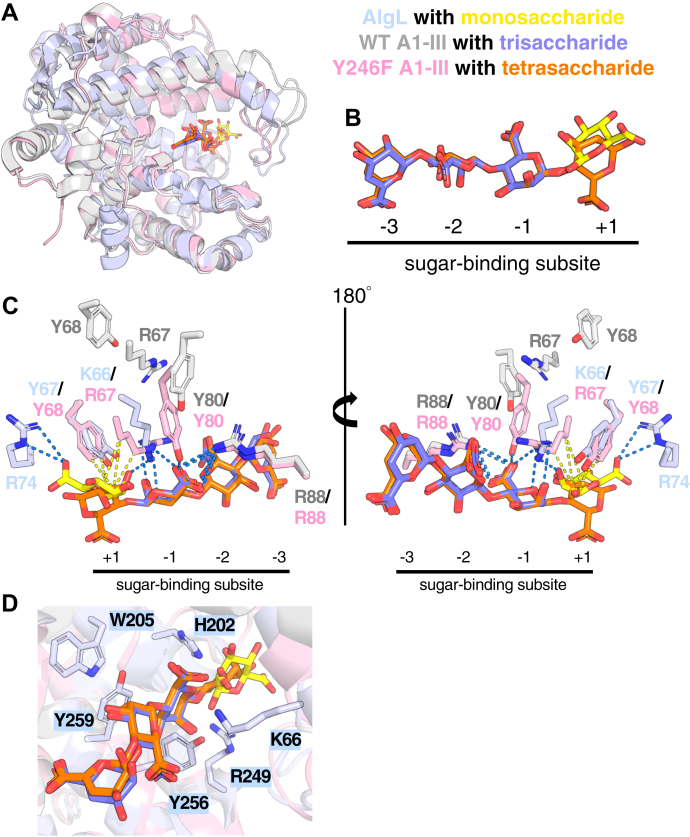
**The polysaccharide family 5 bacterial alginate lyases *Pseudomonas aeruginosa* AlgL and *Sphingomonas* sp. A1-III are structurally similar.***A*, superposition of *P. aeruginosa* AlgL (*light blue*) in complex with a monosaccharide of mannuronate (*yellow*) (PDB: 4OZV), *Sphingomonas* sp. WT A1-III (*grey*) in complex with a mannuronate trisaccharide (*purple*) (PDB: 1HV6) (52), and *Sphingomonas* sp. Y256F A1-III (*pink*) in complex with a mannuronate tetrasaccharide (*orange*) (PDB: 4F13) (53). *B*, ligands bound and their sugar-binding subsite positions in the *P. aeruginosa* AlgL and *Sphingomonas* sp. A1-III structures. *C*, close-up of the lid-loop region of the *P. aeruginosa* AlgL and *Sphingomonas* sp. A1-III active sites. Hydrophobic and hydrogen-bonding interactions are represented by the *dashed yellow* and *blue lines*, respectively. *D*, *P. aeruginosa* AlgL active site residues (*light blue*) chosen for downstream *in vitro* and *in vivo* mutagenic studies, including the proposed catalytic acid and base Y256, the residue that stabilizes the anionic intermediate H202, the lid-loop residue K66, the highly conserved residues W205 and Y259, and R249 involved in neutralizing the C-5 carboxylate group.

To gain further insight into the structure and function of AlgL and identify key residues involved in substrate binding and catalysis, we compared our structure with the known structures of the PL-5 family member, *Sphingomonas* sp. A1-III. Of the five available A1-III structures, two are complexed with polyM oligosaccharides. The WT A1-III in complex with a polyM trisaccharide (PDB: 1HV6) (52) and the Y246F mutant in complex with a polyM tetrasaccharide (PDB: 4F13) (53) were therefore compared with our AlgL-ManA structure. Superposition of our AlgL structure with WT and Y246F A1-III resulted in a C_α_ RMSD of 1.965 Å and 1.895 Å, respectively, highlighting the similarity of the overall tertiary structure of the enzymes (Fig. 3*A*).

A noticeable difference between the three structures involves the position and orientation of the bound ligands (Fig. 3, *A* and *B*). Within the sugar-binding site of WT A1-III, the trisaccharide occupies subsites −3 to −1 (Fig. 3*B*; (52)), while the tetrasaccharide in the catalytically inactive Y246F A1-III mutant structure occupies subsites −3 to +1. Cleavage of the ligand occurs between subsites −1 and +1 (Fig. 3*B*; (53)). Both ligands bind in a similar orientation in subsites −3 to −1 (Fig. 3*B*). Similar to Y246F A1-III, the sugar monomer modeled in our AlgL structure occupies subsite +1 (Fig. 3*B*). However, this monomer adopts a different orientation; a variation that is most likely attributable to the lack of additional conformational restraints that a longer sugar polymer would provide (Fig. 3*B*).

Comparison of the three structures reveals a striking difference in the extended loop that partially encloses the ligand, termed the lid-loop: residues 64 to 85 in A1-III, and 63 to 90 in AlgL (Figs. S2 and 3*A*; (53)). This loop has previously been suggested to undergo an induced-fit motion during alginate binding and catalysis (53). Although the AlgL and A1-III lid-loops differ in size by six residues, both adopt similar conformations (Fig. 3*A*). The conformation of the lid-loop correlates with the presence of a ligand in the +1 subsite, with the lid-loop in the Y246F A1-III and AlgL structures in a more enclosed conformation, while the loop adopts a more open conformation in the WT A1-III structure (Fig. 3, *A* and *B*). A more enclosed orientation of the lid-loop is also observed in the structure of the H192A A1-III mutant in complex with a polyMG tetrasaccharide, which occupies subsites −3 to +1 (53). In our AlgL structure, we found that the N^ζ^ atom of lid-loop residue K66 interacts *via* a hydrogen bond with the hydroxyl group of the C-2 of ManA (Fig. 3*C*). In contrast, in the Y246F A1-III structure, the difference in orientation of the ligand results in residues Y68 forming hydrophobic interactions with the +1 ligand (Fig. 3*C*). In Y246F A1-III, residues R67, Y80, and R88, which are adjacent to the lid-loop, also hydrogen bond to the ligand in the −1 subsite (Fig. 3*C*; (53)). Mutation of residues in the lid-loop region of A1-III, including R67, Y68, and Y80, result in less catalytically active enzymes *in vitro* compared with WT A1-III (53), highlighting the importance of this loop in the enzymatic mechanism. R67, Y68, and R80 in A1-III correspond to K66, Y67, and F85 in *P. aeruginosa* AlgL (Fig. S2).

Examination of the interactions between the protein and ligand in the active site of the WT and Y246F A1-III structures also revealed that Q134, Q138, H245, R306, R312, D314, and R342 directly hydrogen bond to the ligand, while R88, Q134, Y137, W141, N191, R306, and R312 make hydrophobic contacts (52, 53). In the WT A1-III structure, residues W141 and Y249 also form hydrogen bonds with the bound sugar (52). Of the residues that interact with the ligand in Al-III, only residues W141, N191, Y249, and R342 are conserved in AlgL, and these correspond to W146, N201, Y259, and R352, respectively (Fig. S2; (52)). In addition, in A1-III, residue W195, which corresponds to W205 in AlgL, while not directly involved in binding the carbohydrate, is highly conserved across PL-5 alginate lyases (Fig. S2; (52)).

Studies on A1-III have implicated Y246 as both the catalytic acid and base, with H192 stabilizing the anionic intermediate and R239 interacting with the sugar to neutralize the C-5 carboxylate group (52). Mutation of H192 to alanine and Y246 to phenylalanine drastically decreased A1-III enzyme activity *in vitro* (53). These residues are conserved in AlgL and correspond to Y256, H202, and R249, respectively (Fig. S2).

### Mutation of predicted AlgL catalytic site residues abrogates enzymatic activity *in vitro*

Based on our sequence and structural analysis, we chose six residues for downstream *in vitro* and *in vivo* mutagenic studies (Table 2 and Fig. 3*D*). The residues chosen include the proposed catalytic acid and base Y256, the residue that stabilizes the anionic intermediate H202, the lid-loop residue K66, the highly conserved residues W205 and Y259, and the residue involved in neutralizing the C-5 carboxylate group R249. To determine the importance of these residues in alginate degradation, we first assessed the impact of the point mutations using an *in vitro* alginate lyase activity assay with a polyM substrate derived from *P. aeruginosa*. The assay enabled the steady-state kinetic parameters for the enzymatic reaction to be calculated, including the catalytic efficiency (*k*_*ca*t_/*K*_*M*_), for the WT and each mutant enzyme (Table 3). As anticipated, WT AlgL is the most catalytically efficient enzyme with a turnover rate of 15.7 ± 0.274 s^−1^ and catalytic efficiency of 147 ± 10.6 × 10^3^ (s^−1^ M^−1^) (Table 3). We were unable to detect any catalytic activity using this assay for mutants H202A, R249A, R249E, and Y256F (Table 3). The conservative mutant R249K retained approximately 1.5% catalytic efficiency compared with the WT enzyme (Table 3). Mutation of residues implicated in substrate binding, such as K66 and Y259, led to the retention of enzymatic activity, although these mutants were significantly less catalytically efficient than the WT. K66A and Y259F retained approximately 1% and 30% catalytic efficiency, respectively, compared with WT (Table 3). Mutation of the highly conserved residue W205 also resulted in an enzyme with ∼2.5% catalytic efficiency relative to WT (Table 3). However, loss of enzyme activity in this case could be attributable to protein instability or misfolding as the melting temperature for W205F was more than 4 °C less than the WT enzyme (Table S1). Overall, our data demonstrate that mutation of AlgL residues implicated in catalysis, with the exception of the conservative mutant R249K, results in loss of *in vitro* alginate lyase activity, while mutation of residues implicated in substrate binding greatly reduced, but did not abrogate, enzymatic activity.

**Table 2:** Proposed role of *P. aeruginosa* AlgL residues and the point mutants used in this study

Residue	Proposed function	Point mutants studied
K66	Lid-loop residue directly involved in substrate binding	K66A
H202	Neutralizes anionic intermediate of the substrate	H202A
W205	Highly conserved residue indirectly involved in substrate binding	W205F
R249	Neutralizes C-5 carboxylate group on substrate for proton abstraction	R249A, R249E, R249K
Y256	Catalytic acid/base	Y256F
Y259	Directly involved in substrate binding	Y259F

**Table 3:** *Pseudomonas aeruginosa* AlgL reaction steady-state kinetic parameters^a^

AlgL enzyme^a^	*k*_*cat*_/*K*_*M*_ (s^−1^ M^−1^)	*k*_*cat*_ (s^−1^)	*K*_*M*_ (μM)
WT	147 ± 10.6 × 10^3^	15.7 ± 0.274	107 ± 5.80
K66A	1.37 ± 0.141 × 10^3^	0.128 ± 0.00303	93.5 ± 7.44
H202A	Activity not detected		
W205F	3.89 ± 0.350 × 10^3^	1.92 ± 0.0647	494 ± 27.9
R249K	2.23 ± 0.429 × 10^3^	0.322 ± 0.0165	144 ± 20.3
R249A	Activity not detected		
R249E	Activity not detected		
Y256F	Activity not detected		
Y259F	44.5 ± 7.05 × 10^3^	15.8 ± 0.864	354 ± 36.8

a Initial velocities were fitted to the Michaelis–Menten equation.

### A functional AlgL is required for *P. aeruginosa* viability during alginate production

Previous studies reported variable results when alginate biosynthesis is induced in *algL* deletion mutant strains. Absence of the enzyme was shown to cause cell death in *P. aeruginosa* FRD1 (47), while no significant loss of biofilm biomass was observed in *P. aeruginosa* PDO300 (48). To further probe the role of AlgL *in vivo* and its impact on cell viability during alginate biosynthesis, we first generated an *algL* deletion in our *P. aeruginosa* PAO1 Δ*wspF* P_BAD_*alg* strain background that allows for induction of alginate expression using L-arabinose in a high c-di-GMP background (28). In this strain, alginate production is isolated from its native AlgU/T system of regulation and can readily be switched on and off with the addition of L-arabinose to the growth medium. Complementation of the Δ*algL* strain with either WT *algL* or active site point mutants was performed using an integrating plasmid at the chromosomal *attTn7* site with the complemented gene also under the control of an arabinose-inducible promoter, thus allowing for simultaneous expression with other alginate proteins within the operon. To ensure that the results we observed could be correlated with a particular mutation, we first confirmed using Western blot analysis that each AlgL variant is expressed 1 h after induction with 0.5% (w/v) L-arabinose in the complemented PAO1 Δ*wspF* P_BAD_*alg* strains (Fig. S3).

**Figure 4:**
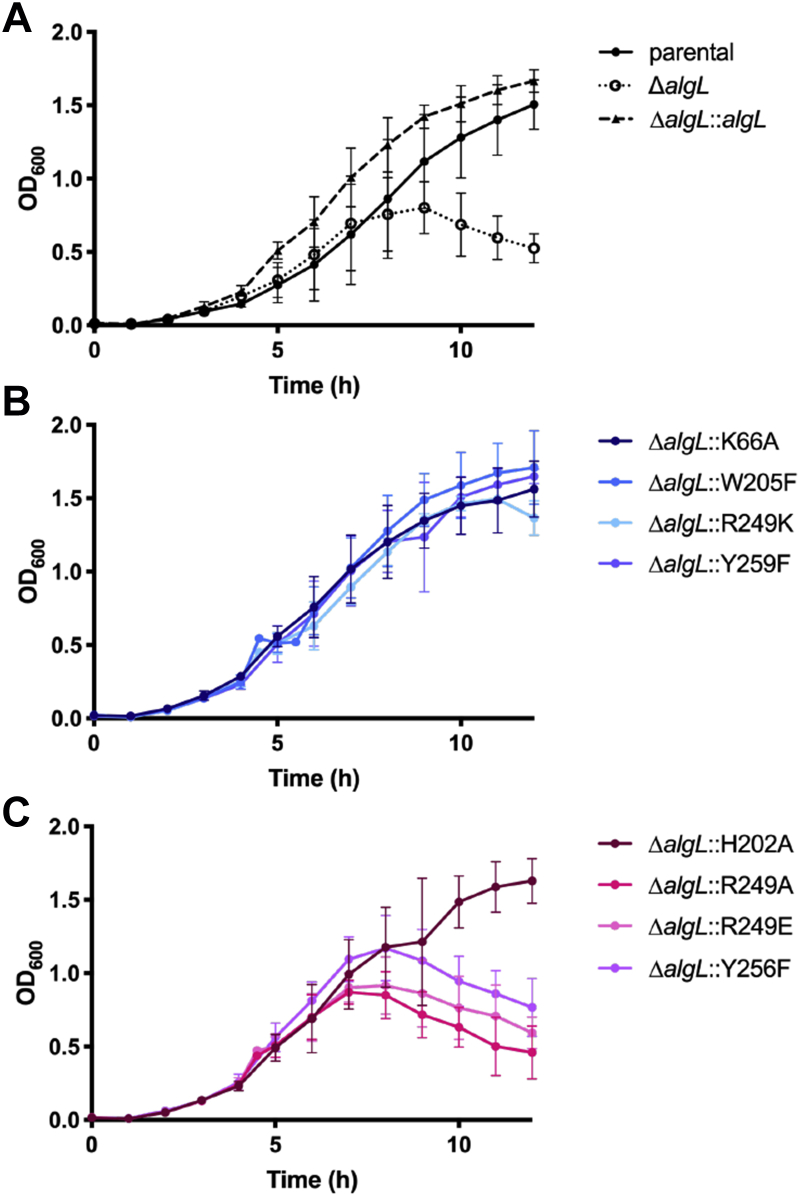
**Growth of *Pseudomonas aeruginosa* PAO1 Δ*wspF* P**_**BAD**_***alg* strains reveals mutation of R249 and Y256 impacts cell viability during alginate production.** After reaching an OD_600_ of approximately 0.5, cells were induced to produce alginate with 0.5% (w/v) L-arabinose. Data points represent averages from three biological replicates with two technical replicates per biological replicate. *A*, control strains. *B*, *ΔalgL* strains complemented with AlgL point mutants that retained alginate lyase enzymatic activity *in vitro*. *C*, *ΔalgL* strains complemented with AlgL point mutants whose alginate lyase enzymatic activity *in vitro* was compromised.

We next investigated whether growth of each strain was compromised in liquid medium (Fig. 4). The optical density of the bacterial culture at 600 nm (OD_600_) was measured every 1 h for 12 h. When strains reached an approximate OD_600_ of 0.500, 0.5% (w/v) L-arabinose was added to the medium. Four hours after induction with L-arabinose, a noticeable growth defect was observed in Δ*algL* compared with the parental strain (Fig. 4*A*). Complementation with WT AlgL (Δ*algL*::*algL*) restored *P. aeruginosa* growth in the presence of L-arabinose, demonstrating that absence of AlgL during alginate production is detrimental to the cell (Fig. 4*A*). When we examined the growth characteristics of AlgL point mutants that retained alginate lyase enzymatic activity *in vitro*, we observed that complementation of the deletion strain with the K66A, W205F, R249K, and Y259F variants exhibited similar growth characteristics as Δ*algL*::*algL* (Fig. 4, *A* and *B*). The effects on growth in the presence of L-arabinose were more pronounced when we examined the AlgL point mutants whose alginate lyase activity was abrogated *in vitro* (Fig. 4*C*). The R249A, R249E, and Y256F strains each displayed a growth defect phenotype (Fig. 4*C*). Interestingly, although we were unable to detect enzymatic activity *in vitro* for the H202A variant, the H202A complemented strain did not display the same *in vivo* growth patterns as the other catalytically inactive mutants (Table 3 and Fig. 4*C*).

Taken together, these data suggest that deletion of *algL* is inhibitory to growth when alginate production is induced with L-arabinose in our genetically engineered strain. In support of this conclusion, we also found that nonconservative mutations of active site residues required for alginate lyase activity, including R249A, R249E, and Y256F, were similarly not tolerated and did not grow under alginate-producing conditions. Mutations that might compromise AlgL’s ability to bind to alginate were found to be tolerated. Thus, our data suggest that the presence of AlgL alone is insufficient to restore viability and that its catalytic activity is required for growth during alginate production in our genetically engineered strain.

### Deletion of AlgL or mutation of its catalytic residues results in abnormal cellular morphology during alginate production

**Figure 5:**
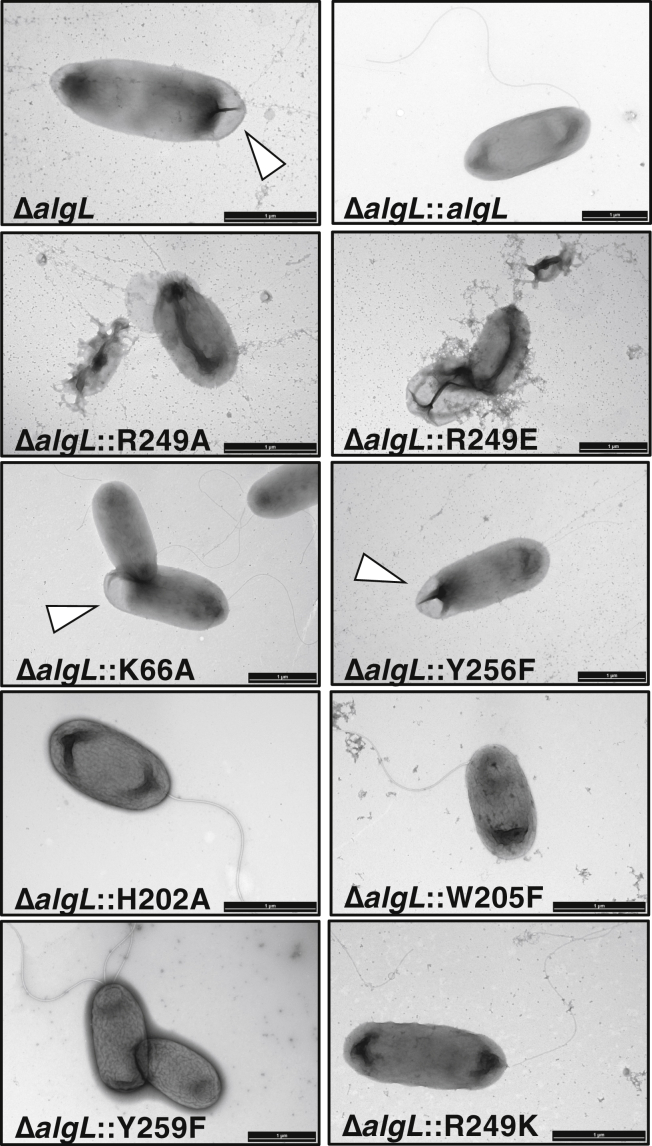
**Transmission electron microscopy images of whole *Pseudomonas aeruginosa* cells after 4 h induction of alginate biosynthesis.***White arrows* indicate cell membrane perturbations. Scale bar is 1 μm.

To further investigate the impact of *algL* mutations on aberrant cell growth, we next sought to assess whether the cell morphology was altered during alginate production. Large zones of separation between membranes after 4 h of alginate production in *P. aeruginosa* FRD1 Δ*algL* and general cell lysis after 6 h have been reported (47). It was hypothesized that these large zones of separation between membranes are indicative of periplasmic alginate accumulation (47). We therefore grew the cells for 4 h post 0.5% (w/v) L-arabinose induction in liquid medium, and transmission electron microscopy (TEM) was used to visualize whole cellular morphologies during alginate production. The micrographs revealed that the Δ*algL* strain and strains for which mutation of the catalytic active site residues resulted in aberrant growth phenotypes, *i.e.*, R249A, R249E, and Y256F (Fig. 4), all displayed abnormal cell morphology compared with the Δ*algL*::*algL* strain (Fig. 5). In particular, *ΔalgL* and Y256F cells have perturbations in the cell membrane (Fig. 5). Cell lysis was observed for R249E and R249A (Fig. 5). Some K66A cells also appear to have membrane perturbations (Figs. S4 and 5). Strains that were observed to grow in alginate-producing conditions, including H202A, W205F, Y259F, and R249K, have cellular morphologies comparable to Δ*algL*::*algL* (Figs. 4*B* and 5). Thus, taken together with our growth data, the TEM images suggest that *algL* mutations that impact function are not tolerated under alginate-producing conditions. In particular, the Δ*algL* strain displays membrane perturbations that we hypothesize are due to the deleterious periplasmic accumulation of alginate.

Overall, the data suggest that AlgL point mutants that have retained some *in vitro* enzymatic activity against polyM do not have a deleterious effect *in vivo*, except for K66A and H202A (Table 3, Figs. 4*B* and 5). Although K66A retains enzymatic activity and the strain does not demonstrate an *in vivo* growth defect, some cells were observed to display aberrant cell membrane phenotypes by TEM (Table 3, Figs. 4*B*, 5 and S4). In contrast, while no enzymatic activity was detected for the H202A variant in our *in vitro* assay, this variant did not display the same growth or cell morphology phenotypes observed for other catalytically inactive mutants (Table 3, Figs. 4*B* and 5), but rather behaved like *ΔalgL*::*algL*.

### The AlgL lid-loop is important for alginate binding and catalysis

To investigate how the H202A and K66A active site mutants might affect enzymatic activity and account for the discrepancies between *in vivo* phenotypes and *in vitro* catalytic activities observed, we determined the structures of the AlgL H202A and K66A variants (Table 1).

Comparison of the K66A variant with WT AlgL revealed that the two structures are highly similar with a C_α_ RMSD of 0.352 Å (Fig. S5). However, residues 66 to 81 within the lid-loop region of K66A AlgL could not be built into the model, suggesting that the lid-loop region in this mutant is flexible (Fig. S5). Similarly, we were unable to observe any corresponding density for the mannuronate substrate soaked into the crystal prior to data collection (Fig. S5). As noted above, K66 interacts with the sugar ligand and appears to contribute to loop enclosure in the WT enzyme (Fig. 3*C*). Mutation of K66 and the resulting flexibility of the lid-loop region suggest that K66 in AlgL is important for priming the enzyme for catalysis by locking the lid-loop in a closed conformation after substrate binding. This could explain the abnormal cellular morphology observed *in vivo* in the K66A strain and the reduced enzymatic efficiency of this mutant variant *in vitro*.

In keeping with its proposed role in neutralizing the anionic substrate intermediate, we found that H202A AlgL was catalytically inactive in our *in vitro* alginate lyase assay and thus were anticipating that this variant would display similar growth and cellular morphology phenotypes to the other inactive variants (Table 3). However, H202A displays a similar growth pattern and cellular morphology to WT (Figs. 4 and 5). When we compared the structure of the mutant with the WT AlgL enzyme, we found that there were strikingly similar with a C_α_ RMSD of 0.062 Å (Fig. S6). There were only minor differences in overall structure of the active sites. Examination of the structure reveals that the orientation of the side chain of K66 is altered, with the N^ζ^ oriented toward the ManA ligand in the WT structure and oriented away from the active site in the H202A structure (Fig. S6). Although no ligands were present during the crystallization process, the lid-loop in the H202A structure adopts a more closed conformation similar to that found in the WT AlgL structure and Y246F and H192A A1-III cocrystal structures (Fig. S7*A*). This conformation appears to be stabilized by hydrogen bonding between R74 and R249 and hydrophobic interactions between K66 and L67 (Fig. S7*B*). To allow for the interaction, R74 in H202A AlgL is oriented toward subsite +1 within the active site, which is occupied by ManA in the WT structure (Fig. S7*B*). In WT AlgL, R74 is oriented away from the active site, suggesting that this residue may be important for anchoring the lid-loop in a closed orientation in the absence of a ligand (Fig. S7*B*). Given the discrepancies observed between *in vivo* growth phenotypes and enzymatic inactivity *in vitro*, and the similarity of the WT and H202A structures, we hypothesize that the H202A mutant is catalytically active, but its activity is below the level that can be detected in our current assay. In support of this, mutation of H192, the equivalent catalytic residue in A1-III, resulted in a large decrease but not complete abrogation of the alginate lyase activity (53).

### AlgL prevents accumulation of alginate within the periplasmic space

**Figure 6:**
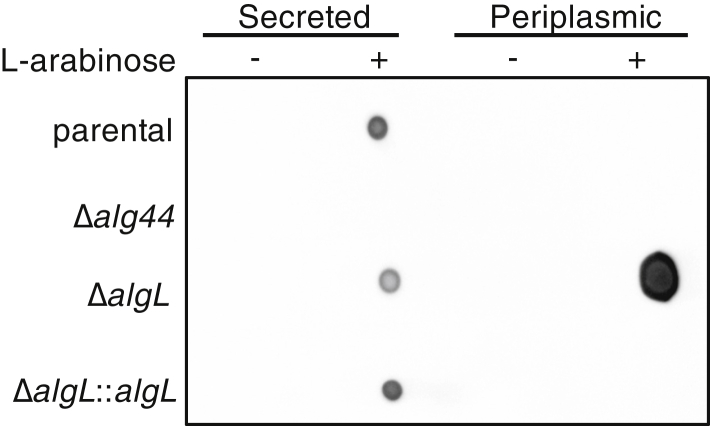
**Alginate is retained in the periplasmic space during alginate production in the absence of AlgL.** Chemiluminescence detection of a dot blot on secreted and periplasmic fractions from *P. aeruginosa* strains using a monoclonal alginate-reactive antibody.

In addition to functioning as an alginate-degrading enzyme, AlgL has also been hypothesized to have a structural role in the biosynthetic complex, interacting with other alginate proteins such as AlgG, AlgK, AlgX, or Alg44 (47). However, protein pull-down and immunoblot assays failed to demonstrate that AlgL is associated with any of these proteins (48). Using a comparable approach, we performed coimmunoprecipitation experiments using AlgL C-terminally tagged with the vesicular stomatitis virus glycoprotein (VSV-G) epitope followed by mass spectrometry analysis and similarly did not enrich for any other alginate proteins or any other protein that was not found in the Δ*algL*::*algL* control sample (WT) (Table S2). Our data reinforce the previous findings and suggest that AlgL does not interact with the alginate secretion complex. These data suggest that AlgL does not need to interact with the rest of the alginate complex to degrade accumulated alginate within the periplasm. To test this hypothesis, we next sought to determine whether the substance we observed accumulating within the cell envelope in our Δ*algL* TEM images is alginate. Periplasmic and secreted fractions of the parental, Δ*algL*, Δ*algL*::*algL*, and Δ*alg44* strains grown in liquid culture were extracted and then analyzed *via* dot blot assays using a commercially available monoclonal alginate antibody. A signal for alginate was detected in the parental, Δ*algL*, and Δ*algL::algL* secreted fractions when polymer production was induced with 0.5% (w/v) L-arabinose (Fig. 6). As expected, alginate is not detected in the *Δalg44* strain, which lacks the c-di-GMP receptor that posttranslationally regulates polymerization (Fig. 6). Alginate was detected in the periplasmic fraction of *ΔalgL*, demonstrating for the first time that loss of AlgL directly results in periplasmic accumulation of the polymer (Fig. 6). Taken together with the Δ*algL* TEM micrographs, these data support the hypothesis that deletion of AlgL results in accumulation of alginate within the periplasmic space during alginate production (Figs. 5 and 6) and suggests that AlgL can function as a periplasmic housekeeping enzyme that maintains cell viability by preventing the accumulation of alginate in the periplasmic space.

### Growth defects due to absence of AlgL during alginate biosynthesis are mitigated by constitutive expression of the AlgU/T regulon

Thus far, using our *P. aeruginosa* PAO1 Δ*wspF* P_BAD_*alg* strain, we have demonstrated that AlgL is not part of the alginate secretion complex and that it degrades polymer that accumulates within the periplasmic space. While the genetic background we used in our *in vivo* studies was crucial for dissecting the role of AlgL, alginate production is normally under the control of the AlgU/T sigma factor. AlgU/T regulates hundreds of genes in PAO1, including genes responsible for mitigating cell wall stress, peptidoglycan biosynthesis, pyoverdine biosynthesis, and lipopolysaccharide biosynthesis (54, 55, 56, 57, 58, 59).

**Figure 7:**
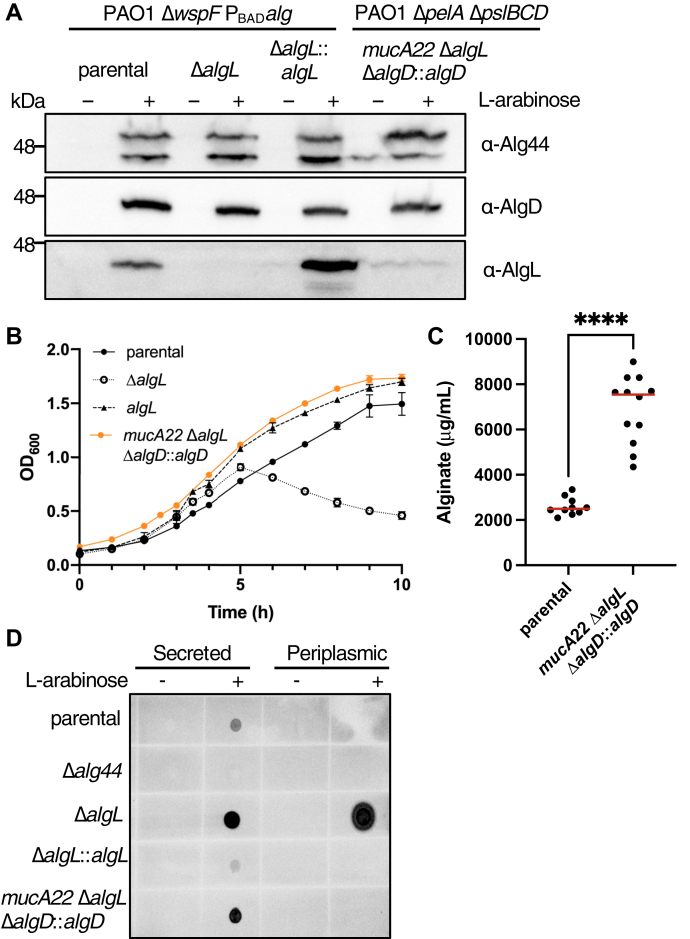
**Expression of the AlgU/T regulon in a Δ*algL* strain background rescues *Pseudomonas aeruginosa* lethality during alginate production.***A*, Western blot analysis of whole cell lysates expressing Alg44, AlgD, or AlgL after 1 h induction with 0% or 0.5% (w/v) L-arabinose using poly-clonal Alg protein specific antibodies. *B*, growth curves of PAO1 Δ*wspF* P_BAD_*alg* (parental), PAO1 Δ*wspF* P_BAD_*alg* Δ*algL* (Δ*algL*), PAO1 Δ*wspF* P_BAD_*alg* Δ*algL*::*algL* (Δ*algL*::*algL*), and PAO1 Δ*pelA* Δ*pslBCD mucA22* Δ*algL* Δ*algD*::*algD* (*mucA22* Δ*algL* Δ*algD*::*algD*). After reaching an OD_600_ of approximately 0.5, cells were induced to produce alginate with 0.5% (w/v) L-arabinose. Values shown represent averages of two biological replicates with two technical replicates for each biological replicate. *C*, quantification of alginate produced over the course of 22 h by parental and *mucA22* Δ*algL* Δ*algD*::*algD* with addition of 0.5% (w/v) L-arabinose to the growth media. Values represent all technical replicates across four separate experiments. *Red lines* represent the median. Statistical analysis was carried out using a Mann–Whitney test: ∗∗∗∗ indicates *p* < 0.0001. *D*, chemiluminescence detection of a dot blot on secreted and periplasmic fraction samples from *P. aeruginosa* strains using a monoclonal alginate-reactive antibody.

In chronic *P. aeruginosa* CF lung infections, alginate production can be induced by a truncation mutation in the AlgU/T antisigma factor, MucA (*mucA*22). This leads to constitutive activation of AlgU/T and thus expression of its vast regulon (25). In our genetically engineered strain, the alginate operon is transcribed in the absence of other genes present in the AlgU/T regulon, which could potentially include genes that can respond to and mitigate the periplasmic stress induced by the accumulation of alginate. We hypothesized therefore that the discrepancies in the current literature regarding the effect of Δ*algL* on *P. aeruginosa* could potentially be attributed to differences in regulation of AlgU/T and the alginate operon in the strains used. To test this hypothesis, we first generated a Δ*algL* mutation in a PAO1 *mucA22* background that is incapable of producing Pel (Δ*pelA*), Psl (Δ*pslBCD*), or alginate (Δ*algD*) (Table S3). To complement the alginate production defect, *algD* was reintroduced at the chromosomal *attTn7* site under the control of an arabinose-inducible promoter (Table S3). Thus, proteins within the alginate operon, with the exception of *algD*, are under the control of their native promoter. We first confirmed that the newly generated PAO1 Δ*pelA* Δ*pslBCD mucA22 ΔalgL* Δ*algD*::*algD* (*mucA22 ΔalgL* Δ*algD*::*algD*) strain was able to express the appropriate alginate proteins by probing for the expression of AlgD, Alg44, and AlgL (Figs. 7*A* and S8). As expected, Alg44 was expressed in the absence of L-arabinose, while AlgD was only expressed after addition of 0.5% (w/v) L-arabinose to the growth media (Figs. 7*A* and S8). Although a faint band can be observed at the expected molecular weight of AlgL, colony PCR of the strain confirms that *algL* is deleted from the genome (Figs. 7*A* and S9). Therefore, we speculate that this band represents nonspecific binding of the antibody to a protein that is expressed as a consequence of constitutive expression of the AlgU/T regulon. We next examined the growth of our parental, Δ*algL*, Δ*algL*::*algL*, and *mucA22 ΔalgL* Δ*algD*::*algD* strains in liquid culture. When strains reached an approximate OD_600_ of 0.500, 0.5% (w/v) L-arabinose was added to the media. As anticipated, the Δ*algL* strain demonstrated an aberrant growth phenotype and the Δ*algL*::*algL* strain grew similarly to the parental strain (Figs. 4*A* and 7*B*). Interestingly, the *mucA22 ΔalgL* Δ*algD*::*algD* strain also grew similarly to the parental and Δ*algL*::*algL* strains, illustrating that loss of *algL* is tolerated during alginate production when the alginate operon is under control of AlgU/T and AlgU/T is free to transcribe all of the genes within its regulon (Fig. 7*B*). Moreover, we found that our *mucA22 ΔalgL* Δ*algD*::*algD* strain is capable of producing at least as much alginate as the parental strain in the presence of 0.5% (w/v) L-arabinose (Fig. 7*C*). After 22 h of alginate production, the parental and *mucA22 ΔalgL* Δ*algD*::*algD* strain reached an average OD_600_ of 2.156 and 2.125, respectively, and produced approximately 2.5 mg/ml and 6.8 mg/ml alginate, respectively (Fig. 7*C*). This suggests that tolerance of an *algL* deletion in *mucA22 ΔalgL* Δ*algD*::*algD* cannot be attributed to a deficiency in alginate production (Fig. 7*C*). Consistent with these results, analysis of the secreted and periplasmic fractions demonstrates that alginate is secreted in the *mucA22* Δ*algL* Δ*algD*::*algD* strain and not retained in the periplasm (Fig. 7*D*).

## Discussion

In this study, by genetically engineering a strain that enables alginate production under the control of arabinose, we have been able to dissect the role of AlgL in *P. aeruginosa* alginate biosynthesis. We found that this enzyme prevents the lethal periplasmic accumulation of polymer, suggesting that the enzyme has a role in homeostasis. Structural analyses of WT *P. aeruginosa* AlgL in complex with ManA, coupled with site-directed mutagenesis and *in vitro* enzymatic assays, enabled the identification of key active site residues involved in alginate binding and catalysis (Figs. 1, 2 and S2; Table 2). We found that mutation of the catalytic site residues R249 and Y256 that abrogate *in vitro* activity was also detrimental for *P. aeruginosa* viability during alginate production (Figs. 4 and 5). Importantly, we demonstrated that any detrimental effects due to the loss of AlgL in *P. aeruginosa* are mitigated by constitutive expression of the AlgU/T regulon (Fig. 7). Collectively, our results define the role of AlgL in alginate biosynthesis and can explain the variations in the *in vivo* phenotypes observed across different *P. aeruginosa* Δ*algL* strains.

Our analyses show that R249 and Y256 are important for AlgL *in vitro* catalytic activity and *in vivo* viability (Table 3 and Fig. 4). PL-5 family alginate lyases are proposed to cleave polyM *via* a *syn* β-elimination reaction where the carboxylate group on C-5 is neutralized and a general base abstracts a proton from C-5 resulting in the elimination of the substituent at C-4 (41, 60). As expected, when the proposed catalytic acid/base residue Y256 was mutated to Y256F, we observed a complete loss of catalytic activity in our *in vitro* alginate lyase assay (Table 3). However, it is still unclear whether Y256 is the only catalytic residue involved in proton transfer during cleavage of ManA residues (53). To cleave ManA substrates in A1-III, R249 is proposed to act by lowering the p*K*_a_ of Y256 to facilitate the abstraction and donation of protons by Y256 (53). In agreement with this hypothesis, no alginate lyase activity was detected for our R249A and R249E mutants, while R249K retained approximately 1.5% catalytic efficiency compared with the WT enzyme (Table 3). However, AlgL was previously shown to also cleave GulA residues *via* an *anti* β-elimination reaction, which could not be mediated by Y256 alone (41). Thus, it was suggested that AlgL could potentially utilize two different catalytic bases for cleavage of each pair of epimeric substrates, as has been reported for chondroitin lyase ABC that cleaves the epimers chondroitin sulfate and dermatan sulfate using a composite active site with two separate histidine residues that act as a general base for each substrate (41, 61). In AlgL, R249 may act as the general base to cleave GulA residues. Although arginine residues are poor catalytic bases because of their relatively high p*K*_a_ (∼12.5) and thus their poor propensity to be protonated at physiological pH, the role of arginine residues as a general base in proton abstraction has been previously reported in other bacterial enzymes including pectate/pectin lyases and fumarate reductase (62, 63, 64, 65). The p*K*_a_ of arginine residues can be perturbed to favor a deprotonated state by surrounding the catalytic arginine with positively charged residues, as is the case in AlgL where K248 and R250 are near R249. This mechanism was previously demonstrated in acetoacetate decarboxylase from *Clostridium acetobutylicum* where the spatial proximity of K116 decreased the p*K*_a_ of the catalytic base K115 to allow for efficient decarboxylation of substrate (66). Furthermore, the mutants K116C and K116N demonstrated significantly reduced enzymatic activity (∼2% of WT activity) while K116R only demonstrated modestly decreased activity (∼20% of WT activity) (66). Thus, R249 may act to reduce the p*K*_a_ of the catalytic acid and base residue Y256 to catalyze the *syn* reaction, while Y256 and R249 function as the catalytic acid and base, respectively, to catalyze the *anti*-reaction.

Herein, we have demonstrated that AlgL functions as a homeostasis enzyme that degrades alginate polymer that accumulates within the periplasmic space (Figure 4, Figure 5, Figure 6). While this role has been proposed previously, the current study is the first to directly probe the periplasmic contents of AlgL-deficient bacteria during alginate production (Fig. 6). The biosynthetic operons of synthase-dependent exopolysaccharide secretion systems each contain an enzyme that can degrade the polymer being synthesized. To date, while it has been hypothesized that the glycoside hydrolases PelA and BcsZ may be required to clear the periplasmic space of Pel and accumulated glucan chains, respectively, no data have been reported to support these functions (67, 68). Of note, in the synthase-dependent *Escherichia coli* poly-*N*-acetylglucosamine (PNAG) system, deletion of the periplasmic bifunctional deacetylase/hydrolase enzyme PgaB did not hinder PNAG synthesis but did prevent its export and resulted in a dramatic expansion of the periplasmic space at the cell poles, the putative site of PNAG biosynthesis (69). Combined with the results of our current study, this adds support to the proposal that glycoside hydrolases/lyases present in synthase-dependent exopolysaccharide secretion systems play a role in bacterial periplasmic homeostasis.

Recognizing the importance of strain backgrounds in studying alginate biosynthesis, we employed the use of our PAO1 *mucA22* Δ*algL* Δ*algD*::*algD* strain to investigate if deletion of *algL* is deleterious in the commonly used *P. aeruginosa mucA22* background (Fig. 7). Unlike the results obtained for our arabinose-inducible Δ*algL* strain, *mucA22 ΔalgL ΔalgD*::*algD* did not demonstrate a growth defect and did not retain alginate in the periplasmic space (Figs. 4 and 7). The critical difference between these two strains is that MucA is truncated in *mucA22* Δ*algL* Δ*algD*::*algD* and therefore unable to regulate AlgU/T (56, 70). Thus, we speculate that there must be one or more genes within the AlgU/T regulon that compensate for the loss of *algL* in a *mucA22* background. This could potentially explain the discrepancies in observed phenotypes across different *P. aeruginosa* Δ*algL* genetic backgrounds such as PDO300, a *mucA22* derivative of PAO1 (48, 71). For example, analysis of the *P. aeruginosa* genome reveals two similar and lesser-known alginate lyases, PA1167 and PA1784, which share 47.0% sequence identity with each other. Their homologue PSPTO_5015 was found to be regulated by AlgU/T in *Pseudomonas syringae* pv. Tomato DC3000 (42, 72, 73). Although PA1167 and PA1784 do not share any significant sequence similarity with AlgL, they share 35.2% and 30.7% sequence identity, respectively, with the homologous PL-7 family alginate lyase A1-II from *Sphingomonas* sp. which preferentially degrades polyMG (42, 72). PA1167 was demonstrated to preferentially degrade polyMG *in vitro*, in contrast to AlgL, which preferentially degrades polyM (42). Given the structure of alginate, it seems ideal for *P. aeruginosa* to have alginate lyases with different substrate preferences to prevent polymer accumulation in the periplasm. It is also possible that, given the lethal consequence of periplasmic alginate retention, *P. aeruginosa* has redundant systems to degrade polymer that is not exported to ensure cell viability. In this instance, AlgL is the primary alginate lyase for alginate degradation and the additional alginate lyases, PA1167 and PA1784, may function as a fail-safe. Moreover, other genes within the vast AlgU/T regulon, such as sugar ABC-transporters may function to recycle cleaved alginates back into the cytosol (74). Thus, the genetic differences between widely used *P. aeruginosa* strains may have nuanced implications on investigating alginate biosynthesis.

Considering the potential significance of a constitutively active AlgU/T in *P. aeruginosa* (75), we might speculate that studies investigating *algL* in the *mucA22* strain FRD1 may demonstrate similar phenotypes as were reported in our *mucA22* Δ*algL* Δ*algD*::*algD* strain and in PDO300 (76). However, in FRD1 with the alginate operon isolated and under the control of an isopropyl β-D-1-thiogalactopyranoside (IPTG)-inducible promoter, a different phenotype was observed (47). Although the alginate operon should theoretically be expressed alongside other AlgU/T genes when IPTG is added to the growth medium, aberrant phenotypes such as cell death and membrane separation were observed in a Δ*algL* mutant as a consequence of alginate production (47). Further study will be required to reconcile the remaining discrepancies between the *mucA22* FRD1 and our *mucA22* Δ*algL* Δ*algD*::*algD* strains. Similarly, in the mucoid Pf201 strain of *Pseudomonas fluorescens*, deletion of *algL* was also found to be detrimental to growth in liquid culture; however, whether the genetic mutation resulting in *P. fluorescens* Pf201 mucoidy is related to *mucA* is unclear (46). Mucoidy can arise from mutations in other genes, such as a truncation of *rsmE* in *Pseudomonas putida* KT2440 and deletion of *kinB* in PAO1 (76, 77). Thus, alginate overproduction can occur in the presence of a WT *mucA* allele, suggesting that there are other mechanisms outside of the AlgU/T regulon, which can induce mucoidy. For example, in a *kinB* mutant, the sigma factor RpoN is required for mucoidy and regulates 926 genes, including genes involved in alginate biosynthesis, carbohydrate metabolism, iron regulation, motility, and quorum sensing (77, 78). As the additional alginate lyases PA1167 and PA1784 are not present in the KinB-RpoN regulon, AlgL would function as the only mechanism by which accumulated alginate in the periplasm can be degraded (79). Thus, mucoidy that occurs independent of the AlgU/T regulon may result in different consequences if *algL* is deleted.

In conclusion, we report the WT structure of AlgL in complex with ManA and identify key active site residues important for alginate binding and catalysis. Our *in vivo* studies demonstrate that AlgL does not associate with other alginate biosynthetic proteins and functions as a periplasmic homeostasis enzyme to clear the periplasm of accumulated alginate to prevent cell lysis. Furthermore, we demonstrate that AlgL is necessary for *P. aeruginosa* viability during alginate production when the *alg* operon is expressed in isolation from the AlgU/T regulon, and that a protein or proteins can compensate for its deletion when the AlgU/T regulon is upregulated. Future studies to investigate the gene(s) responsible for suppressing the effect of an *algL* deletion in a *mucA22* genetic background will improve our understanding of alginate biosynthesis in the context of CF lung infections and provide insight into potential therapeutic target development.

## Experimental procedures

### Bacterial strains, plasmids, and growth conditions

A complete list of the bacterial strains and plasmids used in this study can be found in Tables S3 and S4. All *P. aeruginosa* strains were derived from PAO1 (80). *P. aeruginosa* mutant and complemented strains were generated using allelic exchange and mini-Tn7 mutagenesis, as previously described (81, 82).

Lysogeny broth (LB) contained, per liter of ultrapure water: 10.0 g tryptone, 5.0 g yeast extract, and 5.0 g NaCl. Vogel-Bonner minimal medium (VBMM) was prepared as a 10× concentrate, which contained per liter: 2.0 g MgSO_4_·7 H_2_O, 20 g citric acid, 100 g K_2_HPO_4_, and 35 g NaNH_4_HPO_4_·4 H_2_O, and was adjusted to pH 7.0 and sterilized by filtration. The 10× VBMM solution was diluted to 1× as needed in sterile, ultrapure water. Semisolid media was prepared by adding 1.0% (w/v) noble agar to VBMM, and 1.5% (w/v) agar to LB. Where appropriate, antibiotic selection was added to growth media as follows: for *P. aeruginosa*, carbenicillin (Carb) at 300 μg/ml, and gentamicin (Gen) at 30 or 60 μg/ml, depending on the application as described below; for *E. coli*, Gen at 10 μg/ml, Carb at 100 μg/ml, and kanamycin (Kan) at 50 μg/ml.

### Basic molecular biology methods

Molecular and microbiological techniques were performed according to standard protocols (83). Genomic DNA isolation, plasmid preparation, and DNA gel extraction were performed using nucleotide purification kits purchased from Bio Basic Inc. All primers were purchased from Sigma Aldrich.

### Construction of *P. aeruginosa* chromosomal mutations

In-frame and unmarked deletion of *algL* in *P. aeruginosa* PAO1 Δ*wspF* P_BAD_*alg* was generated using two-step allelic exchange (82). Flanking upstream and downstream regions of the *algL* ORF were amplified and joined by splicing-by-overlap extension PCR (primers listed in Table S4). The upstream forward and downstream reverse primers were tailed with EcoRI and HindIII restriction enzyme sequences, respectively, to enable cloning of the spliced PCR products. The PCR product was gel purified, digested with EcoRI (Thermo Fischer Scientific) and HindIII (Thermo Fischer Scientific) restriction enzymes as per manufacturer’s instructions, and ligated into pEX18Gm using T4 DNA ligase (Thermo Fischer Scientific). The resulting allelic exchange vector, pEX18Gm::*ΔalgL*, was selected for on LB agar supplemented with 10 μg/ml Gen, identified by colony PCR, and verified by Sanger sequencing using M13 forward and reverse primers (Table S4). Deletions of *alg44* and *algD* were similarly constructed (Table S4).

The deletion allele encoded by pEX18Gm::*ΔalgL* was introduced into *P. aeruginosa* PAO1 Δ*wspF* pBAD*alg* or PAO1 Δ*pelA* Δ*pslBCD mucA22* Δ*algD via* biparental mating with the donor strain *E. coli* SM10 (84). Merodiploids were selected on VBMM supplemented with 60 μg/ml Gen. SacB-mediated counter-selection was carried out by selecting for double crossover mutations on no-salt LB (NSLB) agar supplemented with 15% (w/v) sucrose. Unmarked gene deletions were identified by colony PCR with primers targeting the outside, flanking regions of *algL* (Table S4). To confirm the deletion, PCR products were gel purified and sent for Sanger sequencing.

### Construction of mini-Tn7 vectors

The use of the pUC18-mini-Tn7T-Gm for generating single-copy chromosomal insertions at the *attTn7* site in *P. aeruginosa* was previously reported (81). The vector was modified for arabinose-dependent expression of complemented genes, as was previously reported (28). The *araC*-P_BAD_ promoter form pJJH187 was amplified using the primer pair miniTn7-pBAD_F and miniTn7-pBAD_R. The latter contains flanking sequence encoding SmaI, NotI, PstI, and NcoI sites to generate an additional multiple cloning site downstream of the *araC*-P_BAD_ promoter (85) (Table S4). The *algL* ORF was amplified using the primer pair algL_miniTn7_NcoI and algL_miniTn7_SacI, which encode a synthetic ribosome binding site upstream of the start codon (Table S4). The resultant PCR product was cloned into pUCT18T-miniTn7T-Gm-pBAD using NcoI and SacI restriction enzyme cut sites, selected on LB agar with 10 μg/ml Gen and 100 μg/ml Carb, and confirmed by Sanger sequencing using the miniTn7 Seq_F and miniTn7 Seq_R primers (Table S4). Construction of mini-Tn7 vectors with *algD* was similarly constructed (Table S4).

Complemented *P. aeruginosa* strains were generated through incorporation of miniTn7 vectors at the neutral *attTn7* site on the *P. aeruginosa* chromosome *via* electroporation of miniTn7 vectors and the pTNS2 helper plasmid, as previously described (81). Transposon mutants were selected on LB agar supplemented with 30 μg/ml Gen and confirmed by colony PCR using the miniTn7 Seq_F and miniTn7 Seq_R primers (Table S4).

### Growth curve assay

*P. aeruginosa* strains were grown in 5 ml of modified alginate producing (MAP) defined medium containing 100 mM monosodium glutamate, 7.5 mM monosodium phosphate, 16.8 mM dipotassium phosphate, and 10 mM magnesium sulfate supplemented with 30 μg/ml Gen for 16 h overnight at 37 °C shaking (86). The following morning, 2% (v/v) overnight starter cultures were inoculated into 25 ml MAP medium supplemented with 30 μg/ml Gen. Growth was monitored approximately every hour for 12 h by measuring the OD_600_ using an Ultrospec 21000 pro (Biochrom). After reaching mid-logarithmic growth phase at an approximate OD_600_ of 0.5, cultures were subsequently induced with 0.5% (w/v) L-arabinose to induce expression of alginate proteins.

### Imaging of whole cells using transmission electron microscopy (TEM)

For each strain, 5 μl of culture was added to a carbon-coated 200-mesh copper grid and then blotted. Grids were washed once by applying 5 μl of water, and then samples were negatively stained with 2% uranyl acetate. Samples were viewed with a Phillips CM-10 transmission electron microscope operating at 80 kV under standard operating conditions, and images were collected using a SIS/Olympus Morada 11-megapixel charge-coupled device camera.

### *P. aeruginosa* AlgL gene expression in *E. coli*

The nucleotide sequence of *P. aeruginosa* PAO1 AlgL was obtained from the *Pseudomonas* Genome Database (87). AlgL_28–362_ was PCR amplified from genomic DNA, as previously described (88). The primers account for amino acids 28 to 362, thus excluding the N-terminal signal sequence as predicted by SignalP (89). The gene was incorporated into a pET28b vector with a 3′ stop codon for N-terminal His_6_-tag expression, as previously described (88). For each AlgL protein construct, *E. coli* Origami 2(DE3) Competent Cells (Novagen) were transformed with the expression vector and grown in LB Miller broth supplemented with 50 μg/ml Kan at 37 °C. Once the bacterial cell culture reached an OD_600_ of 0.8, protein expression was induced by the addition of IPTG to a final concentration 1 mM. After the cell culture was incubated at 18 °C for 16 h, cells were harvested by centrifugation at 6700*g* for 30 min at 4 °C. Cell pellets were stored at −20 °C until required for protein purification.

### Purification of AlgL protein from *E. coli*

The pellet from 1 l of *E. coli* bacterial culture was thawed and resuspended in 50 ml of cold lysis buffer (50 mM Tris-HCl, pH 8.0, 500 mM NaCl, 1 mM PMSF, 100 mg/ml lysozyme, 100 mg/ml DNase I, with one SIGMA*FAST* Protease Inhibitor Cocktail EDTA-free tablet). Cells were mechanically lysed by three passes through an Emulsiflex C3 (Avestin Inc) at 15,000 psi. The cell lysate was centrifuged at 20,100*g* for 40 min at 4 °C to remove cellular debris. The resultant cell lysate was loaded onto Ni-NTA resin at 4 °C equilibrated with Buffer B (20 mM Tris-HCl pH 8.0, 500 mM NaCl) with 10 mM imidazole. The resin was washed with 30 ml of Buffer B with 30 mM imidazole. The His-tagged protein was eluted off the column using 30 ml of Buffer B with 300 mM imidazole and concentrated by centrifugation with a 30 kDa cutoff Vivaspin Turbo centrifugal concentrator (Sartorius). The concentrated protein was further purified by size-exclusion chromatography using a HiLoad 16/60 Superdex 200 prep-grade column (GE Healthcare) in 50 mM Tris pH 8.0, 150 mM NaCl, and 2% (v/v) glycerol. Finally, the protein was concentrated by centrifugation with a 30 kDa cutoff Vivaspin Turbo centrifugal concentrator (Sartorius) and frozen in aliquots at −80 °C until required. Protein purification was monitored throughout by SDS-PAGE.

### Purification of 6His-tagged AlgL protein for structural studies

The cloning, protein expression, and purification of the native NHis_6_-AlgL_Pa_^28–362^ were previously described (88). The SetMet-incorporated protein was produced using *E. coli* B834 Met^−^ competent cells (Novagen) (90). The SeMet-incorporated NHis_6_-AlgL_Pa_^28–362^ was purified as described for the native protein (88). Mutants of AlgL were constructed with the pET28b::AlgL_Pa_ expression vector as a template using the QuikChange Lightning Site-directed mutagenesis Kit (Stratagene) according to manufacturer’s instructions. Constructs were verified by Sanger Sequencing. All AlgL mutants were expressed and purified as described for the native protein (88).

### Crystallization, data, collection, structure determination, and analysis

To determine the structure of the AlgL-alginate complex, native AlgL was cocrystallized in the presence of 8 mM ManA_3_. The ManA_3_ was prepared by acid hydrolysis as described previously (91). Native AlgL was crystallized as previously described (89). Crystals of SeMet AlgL were obtained in condition 9 from the Crystal Screen Suite (Hampton Research; 0.2 M ammonium acetate, 0.1 M sodium citrate tribasic dihydrate pH 5.6, 30% (w/v) PEG 4000) using 4.7 mg/ml protein. Crystals were cryoprotected by soaking them for 30 s in crystallization solution supplemented with 20% (v/v) glycerol before vitrification by flash freezing. The cryoprotection solution for native AlgL also contained 8 mM ManA_3_. Subsequently, the frozen crystals were stored in liquid nitrogen prior to data collection.

H202A AlgL was crystallized as described for the native protein (89). The best crystals of H202A AlgL were obtained in 0.2 M ammonium acetate, 0.1 M sodium citrate tribasic dihydrate pH 4.6, 26% (w/v) PEG 4000, 0.01 M taurine. Crystals were cryoprotected by soaking for 30 s in crystallization solution supplemented with 20% (v/v) glycerol. The best crystals of K66A AlgL were obtained in 0.275 M K_2_SO_4_, 19% (w/v) PEG 3350, and 0.1 M HEPES pH 6.9. Crystals were cryoprotected by soaking for 10 min with 2 mM mannuronate tetrasaccharide and 20% (v/v) PEG 4000.

For native AlgL and H202A AlgL, data were collected at beam line X29 at the National Synchrotron Light Source (Brookhaven National Laboratory). 360 images of 1° Δφ oscillation on an ADSC Q315 CCD detector with a 200 mm crystal-to-detector distance with an exposure time of 0.4 s per image were collected. The data were processed, integrated, and scaled using the HKL-2000 program suite (92). SeMet SAD data consisting of 360 images of 1° Δφ oscillation on an ADSC Q315 CCD detector with a 300 mm crystal-to-detector distance with an exposure time of 0.5 s per image were also collected, processed, integrated, and scaled using the HKL-2000 program suite (93).

For K66A AlgL, data collection was completed using a D8 Venture X-ray Diffractometer (Bruker AXS) at the Structural & Biophysical Core Facility (The Hospital for Sick Children). In total, 510 total image scans of 1° Δφ oscillation with 60 s exposure times per image were collected using the diffractometer with a Kappa four-circle goniometer and a Photon 100 detector at a crystal-to-detector distance of 75 mm. Data was indexed, integrated, scaled, and merged using the Proteum 2 software (Bruker AXS).

The SeMet-SAD data in conjunction with HKL2MAP (93) were used to locate nine out of 11 selenium sites. Density-modified phases were calculated using SOLVE/RESOLVE (94). The electron density map was interpretable, and the model was built to 75% by PHENIX Autobuild and briefly refined using PHENIX.REFINE (95). The PHENIX AutoMR wizard was used to determine the structure of H202A AlgL by molecular replacement. Additional residues were built using COOT, and the structure was refined with PHENIX.REFINE (95, 96). The native AlgL structure was determined using the PHENIX AutoMR wizard using the H202A AlgL mutant structure as a search model and refined using PHENIX.REFINE. The K66A AlgL structure was solved by molecular replacement with native WT as the starting model using Phaser (97). Translation/Libration/Screw groups used during the refinement were determined automatically using the TLSMD web server (98). The electron density map was of sufficient quality for subsequent manual model building and refinement using COOT and REFMAC5 (99).

### Multiple sequence analysis

Bacterial PL-5 family alginate lyases were identified from the CAZy database (http://www.cazy.org/) (50, 51, 100). Sequences of the six PL-5 family alginate lyases with the established enzyme commission (E.C.) number 4.2.2.3 for mannuronate-specific alginate lyase reactions were taken from GenBank (100). The GenBank accession numbers for the sequences are as follows: *Azotobacter vinelandii* CA (AGK12841.1), *Azotobacter chroococcum* B3 (ASL27682.1), *Cobetia marina* N-1 (BAA33966.1), *P. syringae* 31R1 (SDR80954.1), *P. aeruginosa* (SIP51704.1), *Sphingomonas* sp. A1 (BAB03312.1). Sequences were input into Clustal Omega (101) in FASTA format.

### Structure analysis tools

The electrostatic surface potentials were calculated using APBS Tools (102). Conservation analysis was performed using the ConSurf server (103). All structural figures were generated using PyMOL (The PyMOL Molecular Graphics System, Version 1.2, Schrödinger, LLC).

### Alginate lyase activity assay

The activities of AlgL and its mutants were determined by monitoring the formation of the product of the lyase reaction, unsaturated uronic acids, in a Synergy Neo2 Multi-Mode Plate Reader (BioTek Instruments). The lyase reaction was performed at room temperature in 200 μl of 100 mM Tris-HCl pH 7.5 and 150 mM NaCl, or a similar buffer with 100 mM MES pH 6.0, containing various concentrations of nonacetylated polyM and AlgL. Reactions were allowed to progress for 10 min, measuring the OD of the solution at 240 nm (OD_240_) every 10 s. OD_240_ values were converted to molar concentration using the extinction coefficient of 6150 M^−1^ cm^−1^. Kinetic parameters (*K*_*m*_ and the turnover number *k*_*cat*_) were calculated from initial velocities fitted to the Michaelis–Menten equation. All activity assays were performed in triplicate. Nonacetylated polyM was prepared from *P. aeruginosa* strain FRD462 as described previously (104).

### Periplasmic extractions, alginate purification and detection

A 5 ml starter culture for each strain was grown in MAP medium supplemented with 30 μg/ml Gen for 16 h overnight at 37 °C shaking. The following morning, 2% (v/v) overnight starter cultures were inoculated into 50 ml MAP medium supplemented with 30 μg/ml Gen. Strains were grown to an OD_600_ of ∼0.3 and subsequently supplemented with 0.5% (w/v) L-arabinose to induce expression of alginate protein. After 1 h of growth, the cells were collected by centrifugation and resuspended in 1 ml cold shock buffer (0.2 M Tris-HCl pH 8.0, 0.2 g/ml sucrose, 0.1 M EDTA) and incubated on ice for 20 min. The cells were harvested by centrifugation and resuspended in a periplasmic extraction buffer (10 mM Tris-HCl pH 8.0, 5 mM MgSO_4_, 0.2% (v/v) SDS, and 1% (v/v) Triton X-100) and incubated on ice for 10 min with regular inversion. To collect the periplasmic contents, samples were centrifuged at 2100*g* and supernatants collected. Three volumes of cold 100% isopropanol were added to the bacterial supernatants and periplasmic extraction supernatants and incubated for 16 h overnight at −20 °C to precipitate the exopolysaccharides. The following morning, precipitated material was collected by centrifugation at 6700*g* for 20 min at 4 °C. The supernatants were discarded, and tubes were allowed to air dry for 16 h overnight at room temperature. The following morning, the precipitated material was collected with ultra-pure H_2_O and lyophilized using the VirTis Freeze Dryer Freezemobile 35EL Sentry 2.0 Lyophilizer (SP Scientific) until samples were completely dry. Secreted and periplasmic extractions were resuspended in 1 ml and 500 μl ultra-pure H_2_O, respectively, prior to blotting 4 μl onto a nitrocellulose membrane. The membrane was blocked using 5% (w/v) skim milk powder dissolved in Tris-buffered saline (TBS) (50 mM Tris:HCl pH 7.5 and 150 mM) with 0.1% (v/v) Tween-20 (TBST) for 1 h at room temperature. Blots were washed twice in TBST, and then the membrane was incubated with a *Pseudomonas*-reactive alginate monoclonal antibody (QED Bioscience Inc) at a 1:1000 dilution in TBST at 4 °C for 16 h. Blots were washed five times in TBST and then probed with goat α-mouse horseradish peroxidase (HRP)-conjugated secondary antibody (Bio-Rad) at 1:3000 dilution in TBST for 1 h at room temperature. Blots were washed four times in TBST and once with TBS. Blots were developed using the Super Signal West Pico chemiluminescent substrate from Pierce (Thermo Scientific). Blots were imaged using the ChemiDoc XRS System (Bio-Rad).

### Antibody production

Protein-specific antibodies for Alg44 were produced as described previously (35). AlgL was purified as described in this study, and antibodies were produced as described previously (35). AlgD was expressed and purified as described previously (105, 106), and antibodies were produced as described previously (35).

### Western blot analysis

Overnight cell cultures were grown in MAP medium supplemented with 30 μg/ml Gen for 16 h overnight at 37 °C shaking. Cell culture aliquots were normalized to an OD_600_ of 1.000 and centrifuged at 25,000*g* for 10 min to isolate cell pellets. Cell pellets were combined with SDS-PAGE sample buffer (4% (w/v) SDS, 0.2% (w/v) bromophenol blue, 20% (v/v) glycerol, and 200 mM dithiothreitol) in a 1:1 ratio and boiled at 95 °C for 20 min prior to loading each sample onto a 12% (v/v) polyacrylamide gel. Protein was transferred to a polyvinylidene fluoride membrane for immunoblotting (Bio-Rad). The membrane was stained with Ponceau S (0.1% (w/v) Ponceau S in 1% (v/v) acetic acid) for 5 min. The membrane was washed with water and imaged using the ChemiDoc XRS System (Bio-Rad). The membrane was blocked using 5% (w/v) skim milk dissolved in TBST for 1 h at room temperature. Blots were washed twice in TBST, and the membrane was then incubated with alginate protein-specific antibodies (QED Bioscience) at a 1:1000 dilution in TBST at 4 °C for 16 h. Blots were washed five times in TBST and then probed with goat α-rabbit HRP-conjugated secondary antibody (Bio-Rad) at 1:3000 dilution in TBST for 1 h at room temperature. Blots were washed four times in TBST and once with TBS. Alginate protein bands were detected using the Super Signal West Pico chemiluminescent substrate from Pierce (Thermo Scientific). Blots were imaged using the Chemidoc XRS System (Bio-Rad).

### Purification and quantification of alginate

Twenty-five millliliters of MAP media supplemented with 30 μg/ml Gen, with addition of 0.5% (w/v) L-arabinose, was inoculated with cells from solid media and grown for 22 h at 37 °C shaking. Cells were removed by centrifugation and culture supernatants were collected. To precipitate alginate, 3× volume of cold isopropanol was added to the supernatants and incubated at −20 °C overnight. Precipitated alginates were collected by centrifugation, and excess isopropanol was removed by air drying samples at room temperature overnight. Samples were collected and resuspended in 15 ml ultrapure H_2_O and then lyophilized to dryness using the VirTis BenchTop Pro Freeze Dryer (SP Scientific Products). Samples were resuspended in 1 ml PBS and incubated with 30 μg/ml each DNase I (Bio-Basic) and RNase A (Bio-Basic) overnight at 37 °C. The following day, samples were incubated with 30 μg/ml proteinase K (Bio-Basic) overnight at 37 °C. Samples were dialyzed against ultrapure H_2_O overnight using a 3.5 kDa molecular weight cutoff dialysis membrane (FisherBrand). Samples were collected and lyophilized to dryness using the VirTis BenchTop Pro Freeze Dryer (SP Scientific Products). Samples were assayed for alginate concentration using a colorimetric test for uronic acids with alginic acid from *Macrocystis pyrifera* (Sigma-Aldrich) used as the standard, as was previously described (47, 107).

## Data availability

All the data described are located within the manuscript and the supplemental information. The coordinates and structure factors for WT AlgL and the K66A and H202A mutants have been deposited in the PDB, ID codes 4OZV, 7SA8, and 4OZW, respectively.

## Supporting information

This article includes supporting information (100, 108, 109, 110, 111, 112, 113).

## Conflict of interest

The authors declare that they have no conflicts of interest with the contents of this article.
