# Supplementary material for: The Pseudomonas aeruginosa homeostasis enzyme AlgL clears the periplasmic space of accumulated alginate during polymer biosynthesis
Source: J Biol Chem. 2022 Jan 3;298(2):101560. doi: 10.1016/j.jbc.2021.101560 (PMC8829089; doi:10.1016/j.jbc.2021.101560)
Supplement: Supplemental Figures S1–S9 and Tables S1–S4 [file mmc1.docx]

**Supporting information For**

**The *Pseudomonas aeruginosa* homeostasis enzyme AlgL clears the periplasmic space of accumulated alginate during polymer biosynthesis**

Andreea A. Gheorghita^1,2^, Francis Wolfram^1^, Gregory B. Whitfield^1,2,$^, Holly M. Jacobs^3^, Roland Pfoh^1^, Steven S.Y. Wong^1,2^, Allison K. Guitor^1,%^, Mara C. Goodyear^4^, Alison M. Berezuk^4,#^, Cezar M. Khursigara^4^, Matthew R. Parsek^5^, P. Lynne Howell^1,2*^

^1^Program in Molecular Medicine, The Hospital for Sick Children, Toronto, ON, Canada

^2^Department of Biochemistry, University of Toronto, Toronto, ON, Canada

^3^Molecular and Cellular Biology Graduate Program, University of Washington, Seattle, WA, USA

^4^Department of Molecular and Cellular Biology, University of Guelph, Guelph, ON, Canada

^5^Department of Microbiology, University of Washington, Seattle, WA, USA

Current addresses:

^$^Département de Microbiologie, Infectiologie et Immunologie, Université de Montréal, Montréal, QC, Canada

^%^Department of Biochemistry and Biomedical Sciences and the Michael G. DeGroote Institute for Infectious Disease Research, McMaster University, Hamilton, ON, Canada

^#^Department of Biochemistry and Molecular Biology, University of British Columbia, Vancouver, BC, Canada

**SUPPORTING INFORMATION TABLES**

**Table S1. Melting temperatures of *Pseudomonas aeruginosa* AlgL point mutants as determined by circular dichroism.**

| **AlgL Enzyme** | **T_m_ (°C)** |
| --- | --- |
| WT | 47.93 ± 0.15 |
| K66A | 47.07 ± 0.05 |
| H202A | 48.37 ± 0.09 |
| W205F | 43.57 ± 0.13 |
| R249K | 47.39 ± 0.07 |
| R249A | 47.69 ± 0.05 |
| R249E | 48.34 ± 0.09 |
| Y256F | 46.80 ± 0.09 |
| Y259F | 47.71 ± 0.13 |

**Table S2. Peptides detected by liquid-chromatography mass spectrometry analysis of AlgL-VSV-g co-immunoprecipitation from *Pseudomonas aeruginosa.***

| **Spectral counts** | |  | |  |
| --- | --- | --- | --- | --- |
| **WT** | **AlgL-VSV-g** | **Protein** | **Accession Number** | |
| 0 | 37 | AlgL | AlgL_PSEAE | |
| 49 | 35 | 60 kDa chaperonin | CH60_PSEAE | |
| 69 | 16 | B-type flagellin | FLICB_PSEAE | |
| 28 | 15 | Azurin | AZUR_PSEAE | |
| 14 | 10 | 50S ribosomal protein | RL7_PSEAE | |

**Table S3. Bacterial strains and plasmids used in this study.**

| **Strain** | **Description** | **Reference** |
| --- | --- | --- |
| ***E. coli*** |  |  |
| Origami^TM^ 2(DE3) | Protein expression strain; *∆(ara-leu)7697 ∆lacX74 ∆phoA Pvu*ll *phoR araD139 ahpC gale galK rpsL* F’ [*lac*^+^ *lac*I*^q^ pro*] (DE3) *gor522*::Tn*10 trxB,* Str^R^, Tet^R^ | Novagen |
| TOP10 | Cloning strain; F^-^ *mcrA∆*(*mrr-hsdRMS-mcrBC*) Φ80*lacZ* ∆M15 ∆*lacX*74 ∆*araD*139∆(*ara leu*) 7697 *galU galK rpsL endA*1 *nupG*, Str^R^ | Invitrogen |
| DH5α | Cloning strain; F^-^ Φ80*lacZ* ∆M15 ∆(*lacZYA-argF*) U169 *recA1* *endA1 hsdR17* (r_K_^-^, m_K_^-^) *phoA supE44* λ^-^ *thi-*1 *gyrA96 relA1* | Invitrogen |
| SM10(λpir) | Biparental mating donor strain; *thi thr leu tonA labY supE recA*::RP4-2-Tc::Mu Km λpir, Kan^R^ | Simon *et al*., 1983 |
| ***P. aeruginosa*** |  |  |
| FRD462 | A chemically mutagenized strain of *P. aeruginosa* FRD1 that is incapable of incorporating guluronate residues into alginate, *algG4.* | (103) |
| PAO1 | Wild-type strain | M. R. Parsek |
| PAO1 *∆wspF* P_BAD_*alg* | PAO1 *∆wspF* (in-frame); *araC*-P_BAD_ inserted upstream of *alg* operon | (28) |
| PAO1 *∆wspF* P_BAD_*alg* Gm^R^ | PAO1 *∆wspF* (in-frame); *araC*-P_BAD_ inserted upstream of *alg* operon, Gen^R^ | This study |
| GBW14 | PAO1 *∆wspF* P_BAD_*alg ∆algL* | This study |
| GBW15 | PAO1 *∆wspF* P_BAD_*alg ∆algL,* Gen^R^ | This study |
| GBW16 | GBW14 *attTn7*::miniTn7T-Gm::*araC*- P_BAD_AlgL_Pa_, Gen^R^ | This study |
| SSYW1 | GBW14 *attTn7*::miniTn7T-Gm::*araC*- P_BAD_AlgL_Pa_(H202A), Gen^R^ | This study |
| SSYW2 | GBW14 *attTn7*::miniTn7T-Gm::*araC*- P_BAD_AlgL_Pa_(K66A), Gen^R^ | This study |
| SSYW3 | GBW14 *attTn7*::miniTn7T-Gm::*araC*- P_BAD_AlgL_Pa_(W205F), Gen^R^ | This study |
| SSYW4 | GBW14 *attTn7*::miniTn7T-Gm::*araC*- P_BAD_AlgL_Pa_(Y259F), Gen^R^ | This study |
| SSYW5 | GBW14 *attTn7*::miniTn7T-Gm::*araC*- P_BAD_AlgL_Pa_(Y256F), Gen^R^ | This study |
| AAG1 | GBW14 *attTn7*::miniTn7T-Gm::*araC*- P_BAD_AlgL_Pa_(R249A), Gen^R^ | This study |
| AAG2 | GBW14 *attTn7*::miniTn7T-Gm::*araC*- P_BAD_AlgL_Pa_(R249E), Gen^R^ | This study |
| AAG3 | GBW14 *attTn7*::miniTn7T-Gm::*araC*- P_BAD_AlgL_Pa_(R249K), Gen^R^ | This study |
| PAO1 ∆*pelA* ∆*pslBCD* | PAO1 with deletions of *pelA* and *pslBCD* | Borlee *et al*., 2010 |
| HJ1 | PAO1 ∆*pelA* ∆*pslBCD mucA22* | This study |
|  |  |  |
| HJ2 | HJ1 ∆*algD* | This study |
| AAG4 | HJ2 ∆*algL* | This study |
| AAG5 | AAG4 *attTn7*::miniTn7T-Gm::*araC*- P_BAD_*algD*, Gen^R^ | This study |
| GBW17 | PAO1 *∆wspF* P_BAD_*alg* ∆*alg44* Gm^R^ | This study |
| GBW18 | GBW14 *attTn7*::miniTn7T-Gm::*araC*- P_BAD_AlgL_Pa_-VSV-g, Gen^R^ | This study |
| **Plasmid** | **Description** | **Reference** |
| **Recombinant Protein Expression** | | |
| pET28b | IPTG-inducible expression vector encoding N-terminal hexahistidine tag, a thrombin cleavage site, and an optional C-terminal hexahistidine tag, Kan^R^ | Novagen |
| pET28b::AlgL_Pa_ | pET28b with *P. aeruginosa* PAO1 *algL* corresponding to residues 28-362 fused to a TEV-cleavable N-terminal His6 tag; Kan^R^ | (88) |
| pET28b::AlgL_Pa_H202A | pET28b::AlgL_Pa_ with a H202A mutation in the AlgL gene | This study |
| pET28b::AlgL _Pa_K66A | pET28b::AlgL_Pa_ with a K66A mutation in the AlgL gene | This study |
| pET28b::AlgL_Pa_W205F | pET28b::AlgL_Pa_ with a W205F mutation in the AlgL gene | This study |
| pET28b::AlgL_Pa_Y259F | pET28b::AlgL_Pa_ with a Y259F mutation in the AlgL gene | This study |
| pET28b::AlgL_Pa_Y246F | pET28b::AlgL_Pa_ with a Y256F mutation in the AlgL gene | This study |
| pET28b::AlgL_Pa_R249A | pET28b::AlgL_Pa_ with a R249A mutation in the AlgL gene | This study |
| pET28b::AlgL_Pa_R249E | pET28b::AlgL_Pa_ with a R249E mutation in the AlgL gene | This study |
| pET28b::AlgL_Pa_R249K | pET28b::AlgL_Pa_ with a R249K mutation in the AlgL gene | This study |
| **Allelic exchange** |  |  |
| pEX18Gm | Suicide vector for allelic exchange in *P. aeruginosa*, encodes SacB, Gen^R^ | Hoang *et al*., 1998 |
| pEX18Gm::*∆algL* | pEX18Gm with *P. aeruginosa* PAO1 *algL* cloned between EcoRI and HindIII sites, Gen^R^ | This study |
| pEX18Gm::∆*alg44* | pEX18Gm with *P. aeruginosa* PAO1 *alg44* cloned between EcoRI and HindIII sites, Gen^R^ | This study |
| pDONRPEX18Gm | pEX18Gm containing a HindIII flanked Gateway donor site from PMK2010, Gen^R^ and Cm^R^ | Harrison *et al*., 2020 |
| pDONRPEX18Gm::*∆algD* | pDONRPEX18Gm with *P. aeruginosa* PAO1 *algD* | This study |
| **Complementation analysis** | | |
| pJJH187 | GateWay-compatible plasmid with the *araC* repressor and the P_BAD_ promoter flanked by attL1 and attR5 recombination sites; Km^R^ | (85) |
| pUC18T-miniTn7T-Gm | *aacC1* on miniTn7-based vector with transcriptional terminators at the right end of the Tn7 transposon; Amp^R^, Gen^R^ | (81) |
| pUC18T-miniTn7T-Gm-pBAD | pUC18T-miniTn7T-Gm containing *araC*-P_BAD_ and a downstream MSC (SmaI-NotI-PstI-NcoI) cloned between the HindIII and SacI sites; Amp^R^, Gen^R^ | (28) |
| pTNS2 | Helper plasmid encoding *tnsABCD*, Amp^R^ | (81) |
| p-miniTn7T-AlgL_Pa_ | pUC18T-miniTn7T-Gm-pBAD with *P. aeruginosa* PAO1 *algL* fused to an upstream synthetic ribosome binding site, cloned between the NcoI and SacI sites; Amp^R^, Gen^R^ | This study |
| p-miniTn7T-AlgL_Pa_H202A | p-miniTn7T-AlgL_Pa_ with a H202A mutation in the AlgL gene, Gen^R^ | This study |
| p-miniTn7T-AlgL_Pa_K66A | p-miniTn7T-AlgL_Pa_ with a K66A mutation in the AlgL gene, Gen^R^ | This study |
| p-miniTn7T-AlgL_Pa_W205F | p-miniTn7T-AlgL_Pa_ with a W205F mutation in the AlgL gene, Gen^R^ | This study |
| p-miniTn7T-AlgL_Pa_Y259F | p-miniTn7T-AlgL_Pa_ with a Y259F mutation in the AlgL gene, Gen^R^ | This study |
| p-miniTn7T-AlgL_Pa_Y256F | p-miniTn7T-AlgL_Pa_ with a Y256F mutation in the AlgL gene, Gen^R^ | This study |
| p-miniTn7T-AlgL_Pa_R249A | p-miniTn7T-AlgL_Pa_ with a R249A mutation in the AlgL gene, Gen^R^ | This study |
| p-miniTn7T-AlgL_Pa_R249E | p-miniTn7T-AlgL_Pa_ with a R249E mutation in the AlgL gene, Gen^R^ | This study |
| p-miniTn7T-AlgL_Pa_R249K | p-miniTn7T-AlgL_Pa_ with a R249K mutation in the AlgL gene, Gen^R^ | This study |
| p-miniTn7T-AlgD_Pa_ | pUC18T-miniTn7T-Gm-pBAD with *P. aeruginosa* PAO1 *algD* fused to an upstream synthetic ribosome binding site, cloned between the NcoI and SacI sites; Amp^R^, Gen^R^ | This study |
| p-mini-Tn7T-AlgL_Pa_VSV-G | p-miniTn7T-AlgL_Pa_ with a C-terminal VSV-G tag, Gen^R^ | This study |

**Table S4. Primers used in this study.**

| **Name** | **Sequence (5’ --> 3’)** | |
| --- | --- | --- |
| **Generation of *P. aeruginosa* chromosomal *algL* deletion** | | |
| algLPAO1upF | GGGGAATTCGAAGAGCTTCTACCGCCAGG | |
| algLPAO1upR | TCAACTTCCCCCTTCGCGGCTGATCAGGTGGGACGTTTTCAT | |
| algLPAO1downF | AGCCGCGAAGGGGGAAGTTGA | |
| algLPAO1downR | CGGAAGCTTATCCAGTAGTTGAAGACGGT | |
| **Generation of *P. aeruginosa* chromosomal *alg44* deletion** | | |
| alg44PAO1upF | | GGGGAATTCTGGACCAGCCTGCTCGGCCT |
| alg44PAO1upR | | CAGGGTCACGGCCTTGTTCAGGACGTTGACGGCTGTATTCAT |
| alg44PAO1downF | | CTGAACAAGGCCGTGACCCTG |
| alg44PAO1downR | | GCTGCTGCGGGTCGATTTCC |
| **Generation of *P. aeruginosa* chromosomal *algD* deletion** | | |
| algDPAO1upF | | GGGGACAAGTTTGTACAAAAAAGCAGGCTCAATTTCGCGAGCGGGACAA |
| algDPAO1upR | | GGCCTGGGCAGTGGTGGTGTGCAAACCAAAGATGCTGATTCG |
| algDPAO1downF | | CACACCACCACTGCCCAGG |
| algDPAO1downR | | GGGGACCACTTTGTACAAGAAAGCTGGGTACATCAGGAACACGTGCGAC |
| **Generation of mini-Tn7 vector** | | |
| miniTn7-pBAD_F | GGGAAGCTTTTATGACAACTTGACGGTA | |
| miniTn7-pBAD_R | GGGGAGCTCCCATGGCTGCAGGCGGCCGCCCCG GGCAAAAAAACGGGTATCGAGAAACAGTA | |
| **Generation of AlgL in mini-Tn7 vector** | | |
| algL_miniTn7_Nco1 | TATCCATGGGAGGAGGATATTCATGAAAACGTCCCAC CTGATCCGT | |
| algL_miniTn7_SacI | ACGGAGCTCTCAACTTCCCCCTTCGCGGCTG | |
| **Generation of AlgD in mini-Tn7 vector** | | |
| algD_miniTn7_NotI | GCCCGGGGCGGCCGCGAGGAGGATATTCATGCGAATCAGCATCTTTGGTTTGGGCTATGTCGGTGCAGTATGTGC | |
| algD_miniTn7_SacI | GCATGAGCTCCGGCCCGCTACCAGCAGATGC | |
| **Generation of AlgL point mutants** | | |
| H202A_F | C AAC AAC **GCT** TCC TAC TGG GCG GCC TGG TCG G | |
| H202A_R | CCA GTA GGA **AGC** GTT GTT GAT CTT CTT CAG CGG C | |
| K66A_F | C ACC AGC **GCG** TAC GAA GGC TCC GAT TCG | |
| K66A_R | CT TCG TAC **GCG** CTG GTG AAG ACC AGG CTG | |
| W205F_F | AT TCC TAC **TTT** GCG GCC TGG TCG GTG ATG TCC | |
| W205F_R | GGC CGC **AAA** GTA GGA ATG GTT GTT GAT CTT CTT CAG C | |
| Y259F_F | C TAC CAC AAC **TTT** GCG CTG CCA CCG CTG G | |
| Y259F_R | G CAG CGC **AAA** GTT GTG GTA GGC GAG GGC G | |
| Y256F_F | G CGC GCC CTC GCC **TTC** CAC AAC TAT GC | |
| Y256F_R | GC ATA GTT GTG **GAA** GGC GAG GGC GCG C | |
| R249A_F | C GAA CTC AAG **GCC** CGC CAG CGC GCC CTC GCC TAC CAC | |
| R249A_R | C CCG AAG GAC GGG TTG CTT GAG TTC **CGG** GCG GTC GCG C | |
| R249E_F | C TTC CTG CCC AAC GAA CTC AAG **GAG** CGC CAG CG | |
| R249E_R | CG AAG GAC GGG TTG CTT GAG TTC **CTC** GCG GTC G | |
| R249K_F | C GAA CTC AAG **AAG** CGC CAG CGC GCC CTC GCC TAC CAC | |
| R249K_R | C CCG AAG GAC GGG TTG CTT GAG TTC **TTC** GCG GTC GCG C | |
| **Generation of *mucA22* mutation** | | |
| mucA_F_att | GGGGACAAGTTTGTACAAAAAAGCAGGCTCAATTTCCGCGGCGATAGTG | |
| mucA_R_att | GGGGACCACTTTGTACAAGAAAGCTGGGTAACCGCAAGGACCACTGC | |
| **Generation of AlgL-VSV-g in mini Tn7 vector** | | |
| AlgL_Tn7_C-VSV-G-F | | ATAGATTAGGAAAATGAGAGCTCATGCATGATCGAAT |
| AlgL_Tn7_C-VSV-G-R | | TCATTTCAATATCTGTATAACTTCCCCCTTCGCGGCT |
| **Sequencing** | | |
| M13_F | TGTAAAACGACGGCCAGT | |
| M13_R | CAGGAAACAGCTATGAC | |
| miniTn7 Seq_F | GCGGATCCTAACTGACGCTT | |
| miniTn7 Seq_R | CAAAGGGAATCAGGGATCTTGAAG | |
| mucAseq_F | CGGATCACCGAGCGATAGA | |
| mucAseq_R | AGCTCGAAGCCGTAGCGA | |
| algLPAO1seq_F | TTCCACAAGAACCCGCCGAA | |
| algLPAO1seq_R | ACCACCACGCCGAGGATCAG | |
| alg44PAO1seq_F | GCGGCGCCTGGGCTGGTTCA | |
| alg44PAO1seq_R | GCTGCTGCGGGTCGATTTCC | |
| algDPAO1seq_F | CGGAAAGGCCATCAAGTTG | |
| algDPAO1seq_R | CGGATCACCGAGCGATAGA | |

**Figure S1 Model of the *Pseudomonas aeruginosa* alginate biosynthetic complex.** *A*, Alginate is a carbohydrate polymer composed of 1-4 linked α-L-guluronate (GulA) residues and ß-D-mannuronate (ManA) residues that can be *O-*acetylated (Acetylated ManA). *B*, Schematic of the alginate biosynthetic operon coloured by function. *C*, Schematic of the alginate biosynthetic complex spanning both the inner (IM) and outer (OM) bacterial membranes. Upon binding of c-di-GMP to the PilZ domain of Alg44 (PDB: 4RT0) (35), GDP-mannuronate is transferred to Alg8 for polymerization and transport across the inner membrane. AlgF (PDB: 6CZT), AlgI, AlgJ (PDB: 4O8V) (38), and AlgX (PDB: 4KNC) (36) are required for acetylation of the ManA residues, while AlgG (PDB: 4OZZ) (Wolfram *et al*., 2014) selectively epimerizes unacetylated ManA to GulA (9, 31). AlgK (PDB: 3E4B) (39) is proposed to facilitate alginate export from the porin AlgE (PDB: 3RBH) (40, Tan *et al*., 2014). AlgL (PDB: 4OZV) is an alginate lyase whose function in the system is unclear.

**Figure S2 Multiple sequence alignment of polysaccharide lyase family 5 bacterial alginate lyases with Enzyme Commission # 4.2.2.3 for mannuronate-specific alginate lyase reactions.** Sequences were aligned by the Clustal Omega server (95). Fully conserved residues across the sequences are denoted by an asterisk (*), residues with strongly similar properties are denoted by a colon (:), and residues with weakly similar properties are denoted by a period (.). Residues within the *Sphingomonas* sp. lid-loop region are underlined and bolded. Residues within the *Sphingomonas* sp. active site that are fully conserved in all sequences are highlighted in orange. Residues within the *Sphingomonas* sp. active site that share strongly similar properties are highlighted in yellow. Sequences were taken from GenBank (Clark *et al*., 2016). The GenBank accession numbers for the sequences are as follows: *Azotobacter vinelandii* CA (AGK12841.1), *Azotobacter chroococcum* B3 (ASL27682.1), *Cobetia marina* N-1 (BAA33966.1)*, Pseudomonas syringae* 31R1 (SDR80954.1)*, Pseudomonas aeruginosa* (SIP51704.1), *Sphingomonas* sp. A1 (BAB03312.1). Numbering as per sequences derived from GenBank.

******

**Figure S3 AlgL protein expression in *Pseudomonas aeruginosa* whole cell lysates.** *A*, Western blot analysis of *P. aeruginosa* endogenous expression of AlgL using a polyclonal AlgL-specific antibody. *B*, Ponceau S staining of the membranes from panel *A* demonstrating total protein loaded in each well.

**Figure S4 Transmission electron microscopy images of whole *Pseudomonas aeruginosa* ∆*algL*::K66A cells after 4 h induction of alginate biosynthesis.** White arrows indicate cell membrane perturbations. Scale bar is 2 μm.

**Figure S5 The lid-loop of *Pseudomonas aeruginosa* AlgL is flexible.** Superposition of WT AlgL (light blue) in complex with ManA (yellow) (PDB: 4OZV) and K66A AlgL (orange) (PDB: 7SA8). The lid-loop region of AlgL K66A (dashed orange line) could not be modeled.

**Figure S6 Structure of the H202A AlgL variant is highly similar to WT AlgL.** Superposition of WT AlgL (light blue) in complex with ManA (yellow) (PDB: 4OZV) and H202A AlgL (dark blue) (PDB: 4OZW).

**Figure S7 Comparison of the H202A AlgL variant with wild-type AlgL and A1-III alginate lyase point variants.** *A,* Superposition of H202A AlgL (dark blue) (PDB: 4OZW) with WT AlgL (light blue) in complex with ManA (yellow) (PDB: 4OZV), Y246F A1-III (pink) in complex with a tetrasaccharide (orange) (PDB: 4F13), and H192A A1-III (purple) in complex with a tetrasaccharide (teal) (PDB: 4F10) (52). *B*, Orientation of select substrate binding site residues in H202A AlgL (dark blue) compared to WT AlgL (light blue). Hydrogen-bonding interactions are indicated by the yellow dashed lines.

**Figure S8. Ponceau S stain from blots in Figure 7 demonstrating total protein loaded in each well.**

**Figure S9 Colony PCR of PAO1 ∆*pelA* ∆*pslBCD mucA22* ∆*algL* ∆*algD*::ara-*algD* confirms AlgL was successfully deleted from the chromosome.** Colony PCR using algLPAO1seq_F and algLPAO1seq_R primers from Table S4.

**SUPPORTING INFORMATION METHODS**

*Circular dichroism*

Circular Dichrosim spectra of purified AlgL (0.3 mg/mL) were recorded in 1 mM MES (pH 6.4) and 10 mM NaCl at 20 °C on a JASCO J-810 spectropolarimeter. Melting temperatures were calculated using Prism GraphPad (San Diego, CA).

*Co-immunoprecipitation of AlgL-VSV-G in P. aeruginosa*

Cells were inoculated into 1 L of LB supplemented with 30 μg/mL Gen and 0.5% (w/v) L-arabinose and grown overnight for 16 h at 37 °C shaking. The following morning, cells were collected by centrifugation at 6700 × *g* for 30 min at 4 °C. Cell pellets were transferred to a 50 mL conical tube and resuspended in 50 mL lysis buffer (20 mM Tris-HCl pH 8.0, 100 mM NaCl, 1 mM EDTA, 2% (w/v) Triton X-100, 1.0 mg/mL lysozyme, 0.1 mg/mL DNase, and one SIGMA*FAST*^TM^ Protease Inhibitor Cocktail EDTA-free tablet (Sigma Aldrich). Cells were incubated for 1 h at 4 °C on a rocker. The cell lysates were centrifuged at 20100 × *g* for 40 min at 4 °C to remove cellular debris. Anti-VSV-Glycoprotein-Agarose mouse monoclonal antibody beads (Sigma Aldrich) were resuspended and 60 μL was added to the 50 mL conical tube containing the lysate. The cell lysates were incubated with the agarose beads for 1 h at 4 °C on a rocker. The beads were pelleted by centrifugation at 110 × *g* for 2 min at 4 °C and the supernatant was carefully decanted. The beads were washed three times with 15 mL lysis buffer without added DNase or lysozyme. A final wash was done with 15 mL lysis buffer without added DNase, lysozyme, or Triton X-100. Beads were resuspended in 100 μL 150 mM glycine pH 2.2 and incubated at room temperature for 15 min to eluate the proteins from the agarose beads. The beads were pelleted by centrifugation at 110 × *g* for 2 min at 4 °C and the supernatant was carefully transferred to a microfuge tube for storage and 40 μL of 1 M K_2_HPO_4_ was added. Samples were sent to SPARC BioCentre at The Hospital for Sick Children in Toronto, Ontario, Canada for analysis by liquid chromatography mass spectrometry.
